# Predictors of High Profit and High Deficit Outliers under SwissDRG of a Tertiary Care Center

**DOI:** 10.1371/journal.pone.0140874

**Published:** 2015-10-30

**Authors:** Tarun Mehra, Christian Thomas Benedikt Müller, Jörk Volbracht, Burkhardt Seifert, Rudolf Moos

**Affiliations:** 1 Medical Directorate, University Hospital of Zurich, Zürich, Switzerland; 2 Epidemiology, Biostatistics and Prevention Institute, Department of Biostatistics, University of Zurich, Zurich, Switzerland; School of Medicine, Fu Jen Catholic University, TAIWAN

## Abstract

**Principles:**

Case weights of Diagnosis Related Groups (DRGs) are determined by the average cost of cases from a previous billing period. However, a significant amount of cases are largely over- or underfunded. We therefore decided to analyze earning outliers of our hospital as to search for predictors enabling a better grouping under SwissDRG.

**Methods:**

28,893 inpatient cases without additional private insurance discharged from our hospital in 2012 were included in our analysis. Outliers were defined by the interquartile range method. Predictors for deficit and profit outliers were determined with logistic regressions. Predictors were shortlisted with the LASSO regularized logistic regression method and compared to results of Random forest analysis. 10 of these parameters were selected for quantile regression analysis as to quantify their impact on earnings.

**Results:**

Psychiatric diagnosis and admission as an emergency case were significant predictors for higher deficit with negative regression coefficients for all analyzed quantiles (p<0.001). Admission from an external health care provider was a significant predictor for a higher deficit in all but the 90% quantile (p<0.001 for Q10, Q20, Q50, Q80 and p = 0.0017 for Q90). Burns predicted higher earnings for cases which were favorably remunerated (p<0.001 for the 90% quantile). Osteoporosis predicted a higher deficit in the most underfunded cases, but did not predict differences in earnings for balanced or profitable cases (Q10 and Q20: p<0.00, Q50: p = 0.10, Q80: p = 0.88 and Q90: p = 0.52). ICU stay, mechanical and patient clinical complexity level score (PCCL) predicted higher losses at the 10% quantile but also higher profits at the 90% quantile (p<0.001).

**Conclusion:**

We suggest considering psychiatric diagnosis, admission as an emergencay case and admission from an external health care provider as DRG split criteria as they predict large, consistent and significant losses.

## Introduction

In 2012, Switzerland introduced a diagnosis related groups- (DRG-) based prospective payment system for acute-somatic inpatient care, called SwissDRG. The system is based on the German G-DRG Version of 2008 and is now independently developed from the German system by the SwissDRG AG in Bern. Aims of the introduction were most notably higher efficiency and transparency, as well as an increase in quality of medical care [1]. In Switzerland, DRGs are intended to be comparable in terms of severity of illness, medical conditions and utilization of resources. The relative severity of a DRG, supposedly mirroring the average cost of a DRG case in relation to the average nationwide cost per case, is called the cost weight and is depicted in points. For 2012, a DRG with a cost weight of 1.000 was a DRG with an average severity, a cost weight of over 1.000 indicated a greater than average severity and a cost weight lower than 1.000 a DRG with a severity lower than average. The cost weight of a case is then multiplied by the base rate to obtain the amount which is to be invoiced. Cost weights are calculated based on the total costs on inpatients of the same DRG from a previous billing period. However, two main problems in the calculation of the DRG case weights remain to be resolved. Firstly, the calculation of robust cost weights spanning various billing periods for rare DRGs with very few cases nationwide and a high variation in the costs of these few cases is difficult. Indeed, the small sample population leads to a high volatility in the cost weights for these DRGs from one year to the next: the financial result for these DRGs becomes a lottery. Secondly, only the costs of inliers are considered when calculating cost weights. Hence, cost outliers, which often have a length of stay beyond the high trim point of a DRG or which are grouped into DRGs with insufficient data for exact cost calculation, are excluded from the sample from which the DRG cost weights are calculated. under SwissDRG. Spanish and Belgian studies have shown that high deficit cost outliers account for roughly 5% of cases but produce 11–20% of inpatient costs [2, 3]. According to our data, 10% of cases account for 41% of all costs. Hence, a large proportion of total inpatient costs can be allocated to a small proportion of cases.

Due to the large financial burden incurred by a small percentage of cases, we sought to identify variables predicting cost outliers suitable of becoming DRG split criteria in the future, improving the reimbursement accuracy of the SwissDRG system. Moreover we believe that the novel application of variable selection methods and quantile regression could be of substantial help in identifying new predictors in the pursuit of a more accurate DRG-based reimbursement system.

## Materials and Methods

### Data and definition of high cost cases

Data of 28,893 inpatient cases discharged from the University Hospital of Zurich in 2012 were included into our analysis. Earnings outliers were defined with the interquartile range (IQR) method. Earnings were defined as being the difference between DRG-income and the total cost of a case:

Case earning=DRG-income−total cost

DRG-income was the effective case weight of the case grouped under SwissDRG catalogue version 1.0 multiplied by a base rate of 11,100 CHF plus additional payments (“Zusatzentgelte”):

DRG-income=effective case weight x11,100CHF+additional payments

Total costs are exact total costs of a case and are determined by an accurate, national full cost accounting method with the case as the cost unit [4].

The IQR method defined high deficit cases as those having (negative) earnings less than the first quartile of earnings Q25–1.5 x IQR and high profit cases as those with earnings greater than the third quartile of earnings Q75 + 1.5 x IQR, where QXX is the XX% quantile and IQR the difference Q75—Q25:

$$\begin{array}{ll} & \;\text{High deficit}:\;\text{if case earnings}<\text{Q}25-1.5\;\text{x IQR}\\ \text{IQR rule}:= & \;\text{Normal case}:\;\text{else}\\ & \;\text{High profit}:\;\text{if case earnings}>\text{Q}75+1.5\;\text{x IQR} \end{array}$$

All variables were either diagnoses of the cases coded according to Swiss coding guidelines of 2012 [5] and encoded with ICD-10 GM 2010 [6], the Swiss procedure catalogue CHOP from 2012 [7] and further administrative data. All variables were obtained from the minimal clinical data set and the cost data sent to SwissDRG for the discharges of 2012.

### Logistic regression

Both univariate and multivariate logistic regression models predicting high profit outliers vs. non-outliers and high deficit outlier cases vs. non-outliers were estimated. Results were expressed as odds ratios with p values. Regression coefficients were considered significant at a significance level of p<0.001. We chose such a high significance level due to the large number of variables tested.

### Variable selection

The aim of this manuscript is to determine variables which can reliably help predict high profit and/or high deficit cases. Therefore, the variables should predict substantial differences in the dependent variable with a low alpha level. Variable selection methods can determine the importance of variables in a regression and help compile a shortlist. By assuring significance of the variables with a low alpha level, one safeguards against type I errors. We started with a large number of predictors (95 in total). As we wanted to determine a smaller subset of predictors which predicted large differences in earnings and be suitable for inclusion in the Swiss DRG system, improving its accuracy. For this purpose, we performed two variable selection procedures, namely Random forest [8] and L1 Regularized Logistic Regression (also known as Least Absolute Shrinkage and Selection Operator or LASSO) [9]. Details of both procedures can be found in Hastie et al. [10]. In short, Random forest analysis is a decision tree based selection method which calculates the accuracy of predicting differences in the dependent variable if the chosen independent variable is used as a split criterion for the population. The accuracies are then ranked: a higher rank means that the analysed variable splits the population more accurately into cohorts differing by their dependent or outcome variable. L1 regularized logistic regression or LASSO minimizes the residual sum of squares, the absolute sum of the absolute values of all coefficients having to be less than a constant which is shrunk, forcing a reduction in the value of coefficients, producing coefficients approaching or reaching 0. Lee et al. report a smaller mean squared error than conventional methods as well as an improved handling of multicollinearity next to overall variable selection and coefficients shrinkage as being advantages for the LASSO method, in comparison to multivariate logistic regression analysis with an extended bootstrapping technique and forward variable selection [11]. For Random forest we used the R package Random forest [12], while we used the R package glmnet for the L1 Regularized Logistic Regression [13]. To compare the prognostic accuracy of both variable selection methods with the multivariate regression model, receiver operating characteristic (ROC) curves were used. The area under the curve (AUC) was calculated with corresponding 95% confidence interval. Values between 0.70 and 0.80 can be considered fair, values between 0.80 and 0.90 good, values between 0.90 and 1.0 excellent [14, 15]. For both ROC-curves and AUCs the R package pROC was used [16]. For this purpose, we randomly split the data into a training (20,000 cases) and a test (8,892 cases) set. While the training set was used to derive the regression models, the test set was used to assess the ROC curves and the corresponding AUCs.

### Quantile regression

We chose ten predictors for quantile regression analysis. To be included, the variable had to be selected within the top 15 in both LASSO and Random forest, for at least high profit or high deficit, preferably in both. Moreover, the coefficient of the variable had to be significant in multiple regression analysis (p<0.001). The final decision of including a shortlisted variable in quantile regression analysis was based on the judgement of the authors. We performed a multivariable linear quantile regression predicting five different quantiles Q10, Q20, Q50, Q80, and Q90 of case earnings. Results were expressed as coefficients with corresponding p values. Coefficients of quantile regression estimate the change in a specified quantile of the response variable. Quantile regression can predict absolute values of differences for the dependent variable within quantiles. Details to quantile regression can be found elsewhere [17]. For quantile regression we used the R package quantreg [18].

Regression coefficients were considered significant at a significance level of p<0.001.

### Software

Cases with case information were extracted from the administrative information system (SAP BW) and loaded into the business intelligence software QlikView for further preparation of the dataset, before being exported as a dataset file (Microsoft Excel 2010). All statistical analysis was done using R software [19].

### Ethics

The IRB of the Canton of Zürich explicitly approved our study (KEK-ZH-Nr. 2014–0230). Informed consent was not necessary, as the analysis was done with anonymous, routine clinical and financial data from our hospital. Patient data was anonymized and de-identified prior to analysis.

## Results

### Descriptive statistics

**Fig 1:**
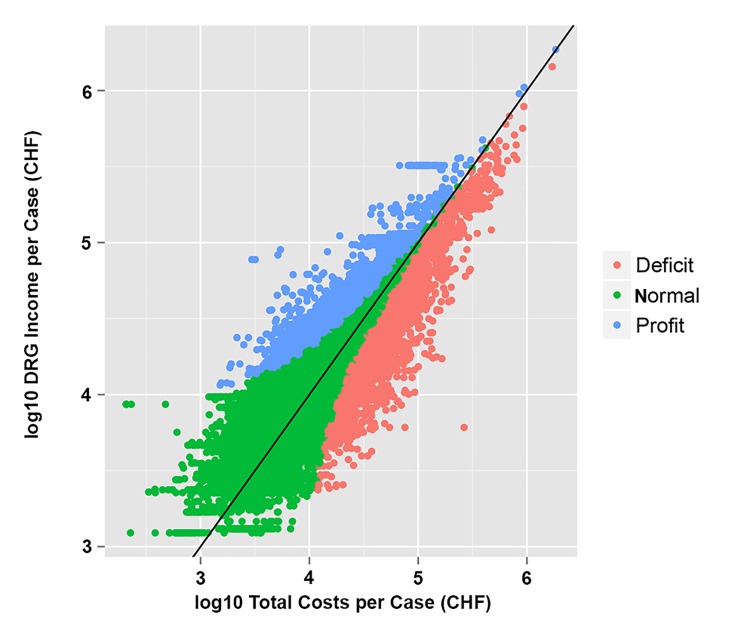
Outlier cases defined for IQR definition (n = 28,892).

28,892 (100%) cases were included in our study. According to the IQR definition of outliers, 2,894 (10%) cases were deficit outliers, 2,029 (7.0%) cases were profit outliers, 23,969 (83%) cases belonging to neither subgroup (Fig 1). The sum of all earnings added up to a deficit of -20,770,419 CHF. Mean earnings were -719 CHF (standard deviation (SD) 1,894 CHF) over all cases, 621 CHF (SD 3,327 CHF) for non-outliers, -30,741 CHF (SD 36,935 CHF) for deficit outliers and 26,277 CHF (SD 31,575 CHF) for profit outliers (p<0.001). Outliers were mostly male (44.7% and 38.7% female cases for the deficit and profit outlier cohort in comparison to 51.9% for the non-outlier cohort). Deficit outliers had a higher average length of stay (LOS) (21.1 days) than the non-outliers (4.5 days and profit outliers (11.8 days). Mortality rates were higher in the deficit outlier (9.3%) and profit outlier (3.4%) cohorts in comparison to the non-outliers (1.4%). The rate of emergency admissions was higher in the deficit outlier (54.1%) and lower in the profit outlier cohort (29.3%) compared to non-outliers (40.3%) (p<0.001).

The mean patient clinical complexity level (PCCL) scores, indicating a calculated index of disease burden per patient based on the amount and constellation of secondary diagnoses, was higher in the outlier cohorts (deficit outliers: 2.9, profit outliers: 2.6; p<0.001) in comparison to the non-outliers (1.1), indicating higher morbidity levels for these sub-populations. The mean case weight of the deficit outlier cohort was lower than that of the profit outlier cohort (3.758 vs. 5.623, p<0.001), in opposite of the mean PCCL scores. The cohort of non-outliers had a mean PCCL score of 0.936. The proportion cases admitted from other inpatient care providers was higher for deficit outliers (20.7%) in comparison to profit outliers (9.2%) and non-outliers (5.7%) (p<0.001). The rate of burns patients was higher in the profit outlier cohort (2.3%) than in the other cohorts (1.1% and 0.2% in the deficit outlier and non-outlier cohorts respectively) (Table 1).

**Table 1:** Sample characteristics. Outliers selected with the IQR method.

	All cases	Non-outliers	Deficit outliers	Profit outliers
Number of cases	n = 28,892	n = 23,969	n = 2,894	n = 2,029
**Earnings in CHF (mean & SD)**	-719 (1,894)	621 (3,327)	-30,741 (36,935)	26,278 (31,576)
**Case costs in CHF (mean & SD)**	18,039 (40,088)	9,795 (11,997)	73,398 (91,834)	36,467 (60,387)
**DRG income in CHF (mean & SD)**	17,320 (34,577)	10,416 (11,859)	42,656 (64,950)	62,745 (76,216)
**Length of Stay (LOS) in days (mean & SD)**	6.7 (10.5)	4.5 (4.7)	21.1 (18.5)	11.8 (15.3)
**Long-stay outliers**	1445	558	860	27
**Long-stay outliers (%)**	5.0%	2.3%	29.7%	1.3%
**Sex (female in %)**	50.2	51.9	44.7	38.7
**Mortality (%)**	2.3	1.4	9.3	3.4
**Emergency admission (%)**	40.9	40.3	54.1	29.3
**PCCL (mean & SD)**	1.4 (1.6)	1.1 (1.5)	2.9 (1.5)	2.6 (1.7)
**Case weight (mean & SD)**	1.548 (3.017)	0.936 (1.057)	3.758 (5.534)	5.623 (6.693)
**Admission from other care providers (%)**	7.4	5.7	20.7	9.2
**Length of Stay ICU in days (mean & SD)**	0.5 (3.5)	0.1 (0.8)	3.5 (9.5)	1.0 (4.5)
**Length of mechanical ventilation in h (mean & SD)**	7.5 (61.8)	1.1 (17.0)	48.2 (160)	25.2 (106)
**Number of visits to the operating theatre (mean & SD)**	0.5 (0.8)	0.4 (0.5)	1.0 (1.7)	0.7 (1.0)
**RBC concentrates (mean & SD)**	0.5 (3.1)	0.1 (0.8)	3.2 (8.7)	1.0 (3.3)
**Thrombocyte concentrates (mean & SD)**	0.1 (1.6)	0.0 (0.6)	0.9 (4.2)	0.5 (2.1)
**FFP concentrates (mean & SD)**	0.1 (3.0)	0.0 (0.3)	1.2 (9.3)	0.1 (1.3)
**Psychiatric diagnosis (%)**	14.5	12.1	33.5	16.8
**Acute myocardial infarction diagnosis (%)**	1.9	1.6	3.4	3.4
**Heart failure diagnosis (%)**	3.4	2.2	9.2	8.8
**Chronic alcohol abus diagnosis (%)**	1.4	1.3	3.1	1.1
**Osteoporosis diagnosis (%)**	2.6	2.1	6.2	3.7
**Pulmonary embolism diagnosis (%)**	0.5	0.3	1.7	0.5
**Pneumonia diagnosis (%)**	2.5	1.4	10.1	3.8
**Diabetes mellitus diagnosis (%)**	9.4	8.2	16.5	13.2
**Acute renal failure diagnosis (%)**	1.6	0.6	8.6	2.9
**Diagnosis of a malignancy (%)**	16.3	14.3	23.5	29.5
**HIV diagnosis (%)**	1.1	1	1.6	1.1
**Surgical or wound complications diagnosis (%)**	3.8	2.4	11.7	8.3
**Postoperative infection diagnosis (%)**	1.2	0.7	4.4	2.7
**Deep vein thrombosis diagnosis (%)**	0.3	0.2	1.5	0.3
**Burns diagnosis (%)**	0.4	0.2	1.1	2.3
**Sepsis diagnosis (%)**	1.6	0.6	7.7	4.7

### Predictors of high deficit cases

ICU stay, PCCL score, respiratory insufficiency, admission from another care provider, psychiatric diagnosis, cerebral infarction and amount of transfused red blood cell units were variables which appeared in the top 15 of both Random forest and LASSO variable selection (Tables 2 and 3). These variables were also significant in the multivariate regression analysis (Table B in S1 Appendix). The appearance of the aforementioned predictors within the selection of two fundamentally different variable selection methods with confirmation of their significance by a third method gave reason to believe in their importance as predictors of high deficit / high profit.

**Table 2:** 15 most important predictors derived from the L1 regularized logistic regression analysis (LASSO) for deficit outliers determined by IQR method. Predictors were ordered by the magnitude of their odds ratio (n = 20,000, training set).

Predictors	Odds ratio
ICU stay (binary)	2.72
Burns (binary)	2.06
PCCL score (range 0.0–4.0)	1.99
Respiratory insufficiency (binary)	1.68
Osteoporotic fracture (binary)	1.62
Dementia (binary)	1.61
Admission from another care provider (binary)	1.56
Epidural hematoma (binary)	1.55
Fracture of the calcaneus (binary)	1.52
Psychiatric diagnosis (binary)	1.46
Cerebral infarction (binary)	1.45
Osteoporosis (binary)	1.43
Plegia (all diagnoses, binary)	1.42
RBC concentrates (number of units)	1.37
Subdural hematoma (binary)	1.35

**Table 3:** 15 most important predictors for deficit outliers defined by the IQR method derived from Random forest analysis. Predictors were ordered by their accuracy (n = 20,000, training set)

Predictors	Accuracy
Number of visits to the operating theatre	37.2
RBC concentrates (number of units)	36.5
LOS at the ICU (in days)	22.9
PCCL score (range 0.0–4.0)	20
Age (in years)	17.4
Reoperation (binary)	14.6
ICU stay (binary)	14.5
Length of mechanical ventilation (in h)	13.3
Admission from another care provider (binary)	12.8
Referral from our hospital to another inpatient care proider (binary)	11.7
Malignant neoplasm (binary)	10.8
Cerebral infarction (binary)	10
Psychiatric diagnosis (binary)	10
Mechanical ventilation (binary)	9.8
Respiratory insufficiency (binary)	9.7

Osteoporosis as well as osteoporotic fractures, burns, dementia, number of visits to the operating theatre and reoperation were variables appearing within the top 15 of either Random forest or LASSO variable selection and were also significant in the multivariate logistic regression analysis.

ICU stay, respiratory insufficiency, admission from another care provider, amount of transfused red blood cell units, number of visits to the operating theatre and reoperations can suggest the occurrence of medical complications and are thus also clinically plausible predictors for high deficit cases. As are a high PCCL score, the coding of psychiatric diagnosis, cerebral infarction, osteoporosis as well as osteoporotic fractures, burns and dementia which, albeit not necessarily indicating a complication, hint at a higher complexity and thus higher resource use.

Epidural hematoma, fracture of the calcaneus, plegia, subdural hematoma, LOS at the ICU, age, length of mechanical ventilation and binary coding of mechanical ventilation, referral from our hospital to another center and malignant neoplasm were variables appearing within the top 15 of either Random forest or LASSO variable selection but were not significant in the multivariate regression analysis.

### Predictors of high profit cases

Burns, leukemia, PCCL score, number of visits to the operating theatre and length of mechanical ventilation were variables appearing within the top 15 of both Random forest and LASSO variable selection analysis (Tables 4 and 5), and were additionally significant independent variables in the multivariate regression (Table D in S1 Appendix).

**Table 4:** 15 most important predictors derived from the L1 regularized logistic regression analysis (LASSO) for profit outliers determined by IQR method. Predictors were ordered by the magnitude of their odds ratio (n = 20,000, training set).

Predictors	Odds ratio
Burns (binary)	16.5
Leukemia (binary)	3.85
ICU stay (binary)	2.59
PCCL score (range 0.0–4.0)	1.82
Supplementary payments ("Zusatzentgelte"—binary)	1.66
Mechanical ventilation (binary)	1.65
Cardiac insufficiency (binary)	1.31
Respiratory insufficiency (binary)	1.24
Malignant neoplasm (binary)	1.18
Lymphoma or plasmocytoma (binary)	1.15
Neopplasm, malignant or of unknown malignancy (binary)	1.15
Sepsis (binary)	1.1
SIRS (binary)	1.09
Length of mechanical ventilation in h	1.07
Number of visits to the operating theatre (binary)	1.06

**Table 5:** 15 most important predictors for profit outliers defined by the IQR method derived from Random forest analysis. Predictors were ordered according to the order of magnitude of their accuracy (n = 20,000, training set).

Predictors	Accuracy
Burns (binary)	35
PCCL score (range 0.0–4.0)	30.9
Emergency admission (binary)	25.5
LOS at the ICU (in days)	19.1
RBC concentrates (number of units)	16.4
Age (in years)	16
Number of visits to the operating theatre	15.7
Reoperation (binary)	15
Length of mechanical ventilation in h	14.5
Lymphoma or plasmocytoma (binary)	14.1
Malignant neoplasm (binary)	13.7
Neopplasm, malignant or of unknown malignancy (binary)	12.8
Mechanical ventilation (binary)	12.4
Referral from our care hospital to another inpatient care proider (binary)	12
Leukemia (binary)	11.4

ICU stay, supplementary payments, respiratory insufficiency, emergency admission, LOS at the ICU, lymphoma or plasmocytoma diagnosis were variables appearing in the top 15 of either Random forest or LASSO analysis, and were significant in the multivariate regression. As for the predictors of high deficit cases, their appearance of within the selection of two fundamentally different variable selection methods with confirmation of their significance by a third method gave reason to believe in their importance.

Mechanical ventilation (binary) and neoplasm diagnosis, either malignant or malignant or of unknown malignancy, were variables appearing in the top 15 of both variable selection methods without being significant in the multivariate regression analysis.

All of the aforementioned predictors indicate case severity, which within the SwissDRG system leads to increased earnings, an elevated PCCL score in its self often triggering a classification of the case into a more highly remunerated DRG.

Cardiac insufficiency, sepsis, SIRS, number of transfused RBC concentrates, age, reoperation and referral from our hospital to another inpatient care provider were variables appearing in the top 15 of one variable selection method only and were not significant in the multivariate regression analysis.

### Quantile regression for earnings

Based on the results from LASSO and Random forest variable selections corroborated with results from the multivariate regression analysis, we chose ten predictors for multivariate linear quantile regression analysis. In particular, ICU stay, PCCL score, admission from another care provider, psychiatric diagnosis and amount of transfused red blood cell units were variables appearing within the top 15 variables selected by both Random forest and LASSO methods within either the high profit or high deficit subpopulations, as well as being significant in the multivariate regression analysis. We further included dementia and osteoporosis for their rising importance due to demographic development. We decided to include emergency admission as due to the high prevalence over all subpopulations and because so far it has not included as a variable within the DRG grouping algorithm [20].

Quantile regression coefficients indicate the expected change in the dependent variable (saldo) for the XX% quantile for a change of one unit of the independent variable: for example a coefficient of -11,009 CHF at Q10 for the binary variable ICU stay, indicates a shift of -11,009 CHF for the 10% quantile for earnings for cases with ICU treatment: at the threshhold of the 10% most underfunded cases, the deficit for cases with ICU treatment is predicted to be 11,009 CHF higher than at the same threshold for cases without ICU treatment. Q10 corresponds to the 10% quantile of lowest earnings (i.e. highest deficits, low outliers) and Q90 to the 10% quantile of highest earnings (i.e. highest profits).

Psychiatric diagnosis (Q10: -4,166 CHF, p<0.001; Q90: -1,378 CHF, p<0.001), admission as an emergency case (Q10: -783 CHF, p<0.001; Q90: -2,158 CHF, p<0.001) and admission from an external health care provider (Q10: -4,004 CHF, p<0.001; Q90: -1,088 CHF, p = 0.0017) were financially relevant predictors with negative regression coefficients for all analyzed quantiles. RBC transfusions (Q10: -5,612 CHF, p<0.0001; Q90: -1.5 CHF, p = 1.00) was a predictor for lower earnings in all quantiles, with a strong association in the 10% quantile and a weak association in the 90% quantile. The ICU-related predictors ICU stay (Q10: -11,009 CHF, p<0.0001; Q90: 4,547 CHF, p<0.001) and mechanical respiratory assistance (Q10: -8,557 CHF, p<0.001; Q90: 9,367 CHF, p<0.001) as well as PCCL score (Q10: -2,038 CHF, p<0.0001; Q90: 1,876 CHF, p<0.001) predicted lower earnings at the 10% quantile and higher earnings at the 90% quantile. Burns predicted higher earnings for the 90% quantile (Q10: -91.3 CHF, p = 0.90; Q90: 18,900 CHF, p<0.001). Osteoporosis (Q10: -7,793 CHF, p<0.001; Q90: 424 CHF, p = 0.52) predicted lower earnings at the 10% quantile (Table 6).

**Table 6:** Quantile regression of ten selected predictors for earnings (n = 20,000, training set).

Predictors	Q10	Q20	Q50	Q80	Q90
**ICU stay (binary)**	-11,009 CHF (< 0.0001)	-6,680 CHF (< 0.0001)	-1,335 CHF (0.0003)	3,127 CHF (< 0.0001)	4,547 CHF (< 0.0001)
**Mechanical ventilation (binary)**	-8,557 CHF (< 0.0001)	-4,978 CHF (< 0.0001)	-949 CHF (0.07)	5,645 CHF (< 0.0001)	9,367 CHF (< 0.0001)
**Dementia (binary)**	-5,858 CHF (0.02)	-4,676 CHF (< 0.0001)	-1,707 CH (0.0028)	-811 CHF (0.04)	-1,675 CHF (0.10)
**PCCL score (score range 0.0–4.0)**	-2,038 CHF (< 0.0001)	-938 CHF (< 0.0001)	78 CHF (0.0003)	964 CHF (< 0.0001)	1,876 CHF (< 0.0001)
**Psychiatric diagnoses (binary)**	-4,166 CHF (< 0.0001)	-2,432 CHF (< 0.0001)	-1,119 CHF (< 0.0001)	-1,259 CHF (< 0.0001)	-1,378 CHF (< 0.0001)
**RBC concentrates(number of transfused units)**	-5,612 CHF (< 0.0001)	-4,165 CHF (< 0.0001)	-2,044 CHF (< 0.0001)	-927 CHF (0.0001)	-1 CHF (1.00)
**Burns (binary)**	-91 CHF (0.90)	-205 CHF (0.88)	5,426 CHF (0.0008)	1,7397 CHF (< 0.0001)	18,900 CHF (< 0.0001)
**Admission from another care provider (binary)**	-4,004 CHF (< 0.0001)	-3,001 CHF (< 0.0001)	-1,411 CHF (< 0.0001)	-970 CHF (< 0.0001)	-108 CHF (0.0017)
**Emergency admission (binary)**	-783 CHF (< 0.0001)	-788 CHF (< 0.0001)	-682 CHF (< 0.0001)	-1153 CHF (< 0.0001)	-2,158 CHF (< 0.0001)
**Osteoporosis (binary)**	-7,793 CHF (< 0.0001)	-4,291 CHF (0.0003)	-455 CHF (0.10)	-35 CHF (0.88)	424 CHF (0.52)

Notes: the selection of the predictors was based on the judgement of the authors, influenced by the results from the two predictor selection methods (L1 regularized regression and Random forest). Q10, Q20, Q50, Q80 and Q90 denote the 10%, 20%, 50%, 80% and 90% quantiles. The results shown are the obtained regression coefficients and the corresponding p values.

## Robustness

We decided to base the variable selection on two established methods, each based on a different selection approach. The LASSO method is a linear regression method proposed by Tibshirani in 1996 [9] which in addition to a shrinkage of ordinary least squares for the error term also poses a constraint on the sum of the absolute values of the regression coefficients, some of which are shrunk to 0. On the contrary, Random forest is a method based on decision tree learning proposed by Breiman [8]. Both methods have found their way into health care research [21]. Before including a variable in the quantile regression analysis, the variable had to be selected within the top 15 in LASSO or Random forest, preferably in both, for at least high profit or high deficit, also preferably in both. Moreover, the coefficient of the variable had to be significant in multiple regression analysis with a low significance threshold of p<0.001, safeguarding against a “false hit” (false rejection of the null hypothesis).

Before including a shortlisted variable in quantile regression analysis, the authors checked for relying on professional experience as to financial relevance and coding reliability of the predictor.

**Fig 2:**
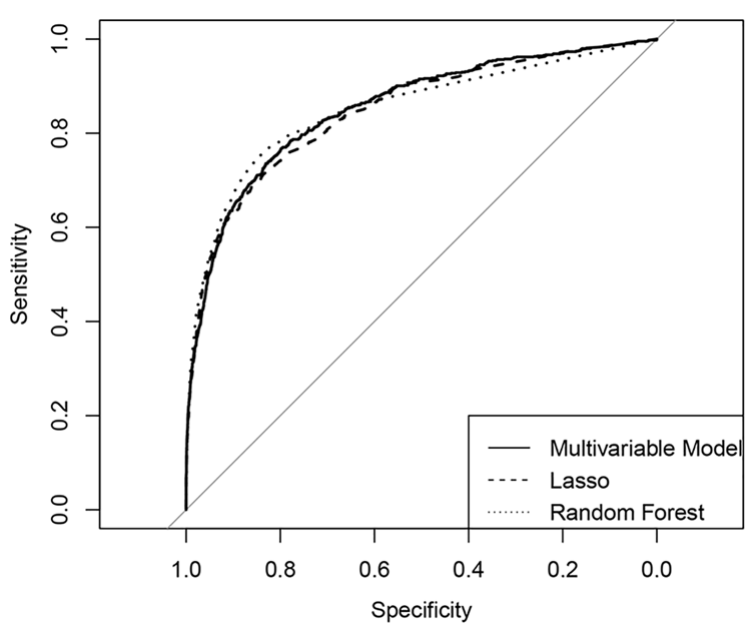
ROC-curves for the multivariate regression model and the two variable selection methods for the prediction of deficit outliers (outlier selection with IQR method) (n = 8,892, test set).

**Fig 3:**
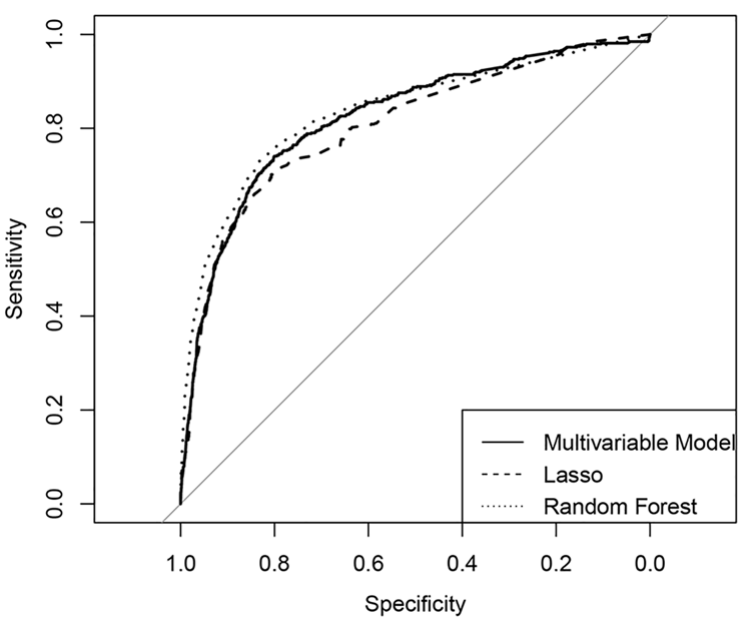
ROC-curves for multivariate regression model and the two variable selection methods for the prediction of profit outliers (outlier selection with IQR method) (n = 8,892, test set).

We tested the fit of the multivariate regression model, Random forest and LASSO graphically with receiver operating characteristic curves (ROC) (Figs 2 and 3) as well as numerically by comparing the area under the curve (AUC) for all three ROCs in the prediction of deficit and profit outliers respectively (Table 7). AUC values where >0.80 for all three models for both variable predicting high deficit and variables predicting high profit cases. The high AUC values for the ROC curves (>0.80) in all methods indicate a high performance/accuracy of the models as to their true positive and false positive rates [14, 15].

**Table 7:** Prognostic accuracy of the multivariate regression model and the two variable selection models for the predictors of deficit and profit cases. Outliers were selected with the IQR method. Results are given as area under the curve (AUC) for a receiver operating characteristic (ROC) curve and the corresponding 95% confidence interval (CI) (n = 8,892, test set).

	Deficit Outlier Model		Profit Outlier Model	
Methods	AUC	95% CI	AUC	95% CI
Multivariable Model	0.87	[0.85, 0.88]	0.83	[0.81, 0.85]
LASSO	0.86	[0.85, 0.88]	0.81	[0.79, 0.83]
Random forest	0.86	[0.85, 0.87]	0.83	[0.81, 0.85]

We compared our selected variables to those obtained with the same data mining methods, whilst changing the outlier definition from an IQR-definition to a 40% deviation of the DRG income minus total costs in relation to total costs (S1 Fig). There was an overlap in the determined variables predicting high deficit as well as high profit (S2 Appendix). However, the fit of the Random forest, LASSO and multivariate regression models was less good with an outlier selection based on % deviation of DRG earnings to costs (S2 and S3 Figs, Table J in S2 Appendix). The IQR definition considers the absolute amount of case earnings/deficits, not the % of the earnings in relation to total costs. This can explain the fact that in the IQR definition, the frequency of outliers in comparison to more costly cases is far lower, as the amount of lower cost cases usually are also on average less highly reimbursed.

Data mining techniques such as the aforementioned variable selection methods as well as clustering high deficit, high profit and non-outliers, can help improve efficiency of statistical analysis, especially of large datasets. However, these techniques can not per se assure validity of the results or coherence of the conclusions drawn. We are aware that one limitation of the study is the quality of the data itself. Nonetheless, internal and independent external revisions assure a high level of data quality. Moreover, our sample is relatively large. Finally, the authors cross-checked the results to make sure they made clinical and economic sense befor drawing conclusions or proceeding with the analysis.

We are aware that our data is from one university center only and that the cross-section of cases can not be considered representative on a national level. However, the aim of the study was to determine novel predictors for high deficit and high profit cases which could be assessed for inclusion in the algorithms of the Swiss DRG system, as to improve its accuracy of predicting reimbursement rates for outlier cases. As university centers are strongly affected by financial imbalances due to outlier cases, the approach of using datasets from university hospitals is not misguided, although the results require validation on a national level.

## Discussion

A prospective payment system such as SwissDRG is designed to reimburse a medical service by remunerating average costs rather than individual costs incurred for comparable services. This is supposed to encourage efficient use of resources and well organized clinical pathways of patient care. Therefore, in principal, a prospective payment system can be deemed fair if the average costs of cases of a DRG are covered by the premium, as stated by Jencks and Dobson in 1987 [22]. However imbalances do arise when higher costs for a care provider incur systematically, for example due to a different patient population or care infrastructure required by an expansive health care mandate. Such is the case of tertiary, mostly university care centers [3]. We therefore wanted to look for additional predictors which could help improve the grouping algorithm by more accurately identifying outlier cases.

The handling of financial outliers is a challenge in every DRG system. In general, outliers are excluded from the calculation of reimbursement rates as not to distort the premiums for “average” cases within a certain DRG. [1] This leads to a systematic underfunding of outlier cases in a standard DRG setting. Hence, most countries have adopted some sort of outlier-specific supplemental reimbursement. For example, cases reimbursed under Medicare in the United States receive an additional payment if costs exceed a pre-determined cost threshold [23]. The hight of the payment is determined by multiplying the difference between the threshold and the actual case costs by a factor, called marginal cost factor, equal to 80% for most DRGs [24]. Most European countires finance outliers based on a per diem charge over a certain length of stay threshold, as cost data are not available for all cases [1]. This is also the case in Switzerland [25]. However, sofar the problem of financial outliers has not been resolved [26–28]. One of the reasons could be that improvements are solely sought within the system, improving the grouping algorithm and re-distributing the cost weights, but not searching for additional parameters which might improve the predictive accuracy.

High profit/deficit cases and quantiles were unconditionally defined in all 28,893 cases, as we wanted to search for novel predictors for high profit/deficit cases beyond already known classificatory variables. We chose to perform a quantile regression, as it can predict absolute values of differences for the dependent variable (earnings) within quantiles i.e. within cases with a high deficit (Q10) or high profit (Q90), depending on the values of the independent variables included. Predictors which have large, consistent and significant coefficients in all quantiles of the quantile regression can predict differences over the entire distribution of cases and are therefore especially well-suited to be included in DRG classificatory algorithms. Psychiatric diagnosis, admission as an emergency case and admission from an external health care provider predicted a higher deficit with negative regression coefficients for all analyzed quantiles. Indeed, a regression coefficient of -4,166 CHF for the 10% quantile of earnings indicates that at the aforementioned quantile, a psychiatric diagnosis would predict earnings to be 4,166 CHF less than those of a case without a psychiatric diagnosis (p<0.001). At the 90% quantile, i.e., cases with high profits, a psychiatric diagnosis still predicted 1,378 CHF less earnings in comparison to cases without a psychiatric diagnosis (p<0.001). The difference in predicted earnings for admissions from another inpatient care provider was similar: a direct referral predicted 4,166 CHF less earnings at the 10% quantile (p<0.001) and 1,378 CHF less earnings at the 90% quantile (p = 0.0017). Emergency admissions predicted significantly lower earnings at the 90% quantile (-2,158 CHF, p<0.001) whereas the difference for cases at the 10% quantile was lower(-783 CHF, p<0.001). The latter difference may be due to the higher discriminatory effect of the variable amongst high profit outliers, as in this subpopulation only 29.3% of the population was admitted as an emergency, in comparison to 54.1% of high deficit outliers or 40.3% of non-outliers. We were surprised that burns predicted higher earnings for cases which were favorably remunerated (Q10: -91 CHF, p = 0.90; Q90: 18,900 CHF, p<0.001). Osteoporosis predicted large and significant differences in cases with low earnings (Q10: -7,793, p<0.001). Osteoporosis may be a good predictor for underfunded cases by serving as a marker for high morbidity and patient frailty. ICU stay predicted a significant difference of -11,009 CHF for cases at the 10% quantile for earnings (p<0.0001) and of 4,547 CHF at the 90% quantile (p<0.001): at the bottom, cases with intensive care treatment are even more underfunded than those without ICU treatment. At the top, they are more overfunded.

ICU stay, PCCL score, respiratory insufficiency, admission from another care provider, psychiatric diagnosis, cerebral infarction and amount of transfused red blood cell units were variables which appeared amongst the top 15 of two different selection methods and were also significant within the multivariate logistic regression analysis, predicting a high loss. Burns, leukemia, PCCL score, number of visits to the operating theatre and length of mechanical ventilation were variables which appeared amongst the top 15 of two different selection methods and were also significant within the multivariate logistic regression analysis, predicting a high profit.

We found it plausible that ICU stay, respiratory insufficiency, admission from another care provider, psychiatric diagnosis, cerebral infarction and amount of transfused red blood cell units predicted a high deficit, as they can be associated with the occurrence of medical complications, which are financially penalized within a prospective payment system. Oncological cases were well funded in the Swiss DRG system 2012 and a high PCCL score often classifies a case within a more highly remunerated DRG, so that did not surprise us that these two variables were identified as important predictors for high profit cases. We were surprised that number of visits to the operating theatre and length of mechanical ventilation were variables predicting high profit as well as high deficit, as these variables would, from a medical point of view be associated with cases with complications. PCCL score indicates a higher case severity, based on the type and number of coded diagnoses. The appearence of this variable as a predictor for high deficit and high profit cases may suggest that the variation of actual incurred costs for very severe cases is high, albeit fixed reimbursement premiums. Hence the Swiss DRG system is not able to accurately predict expected average costs for the most severe cases. We found burns to be amongst the most relevant predictors for profit cases. 124 burns patients were included in the patient subset, including severe as well as non-severe burns. The cumulative earnings of all burns patients amounted to a loss of 113,238 CHF with an average loss per case of 913 CHF. Of the 124 cases, 44 were found in the non-outlier cohort (total earnings: 98,964 CHF, average earnings: 2,249 CHF), 33 cases were included into the deficit cohort (cumulative earnings: -1,616,600 CHF, average earnings: -48,987 CHF) and 47 cases were included into the profit cohort (cumulative earnings: 1,404,397 CHF, average earnings: 29,881 CHF). Even though quantile regression yielded burns as an importantpredictor for increased earnings and both variable selection methods showed burns to be one of the most important variables associated with high profit cases, revenue of burns cases seem to be extremely variable: of the 124 burns cases included, roughly two thirds were outliers. Hence Swiss DRG reimbursement system needs significant improvement in the grouping algorithm and reimbursement rate calculation for burns cases.

Recently, the German institute responsible for the development of the German DRG System, Inek, published a report on cost outliers [26]. They found that considering profit and deficit outliers, the financial burde of deficit outliers overweighed the surplus of profit outliers. Moreover, the financial burden was unevenly distributed between the hospitals, with 167 hospitals profiting financially and 80 having to shoulder a deficit. The burden was especially high for tertiary care centers. Some suggestions for improvement were a re-weighting of especially high intensity ICU care, re-classification of cases with minimally invasive cardiac procedures on more than one heart valve and an expansion of supplementary payments for specific procedures ore medications, for example for the chemotherapeutic agent decitabine. These suggested changes took effect 2015. In Switzerland, the planned changes for the year 2016 include an upgrading of the cost weights for pediatric cases [29]. However, our center does not expect an improvement for financial outliers in general. Our opinion is shared by the Swiss Medical association [30].

Apart from length of stay, Pirson et al. found treatment in an ICU, unplanned admission, major or extreme severity of an illness and presence of social factors to be associated with high resource use outliers [31]. Pirson et al. found that unplanned or emergency admission, ICU treatment and severity of illness, were relevant predictors for cost outliers. Our results showed very similar variables were also important predictors for earnings outliers. This can be explained by the fact that in a DRG based reimbursement system with premiums calculated on the basis of average costs of previous billing periods, cases which incur high costs also receive high premiums. However, due to the amount of actually incurred costs and revenue earnings in absolute terms, small deviations percent-wise of costs from the revenue received rapidly lead these cases to be either highly profitable or unprofitable in real terms. Bekeleis et al. identified number of admission diagnoses and procedures, hospital size and region, and patient income as predictors of cost in patients undergoing cerebral aneurism clipping, apart from length of stay [32]. MaWHinney et al. found urgency/emergency admissions topredict longer length of stay and higher inpatient cost for a cohort of 2,481 cardiac patients. Apart from disease severity indicies, age and gender, Omachonu et al. found mortality, ethnicity, martial status, emergency admission and admission from within a hospital or from another hospital to be important predictors for inpatient length of stay [33] and can therefore be assumed to also be important predictors for inpatient costs. Emergency admissions as well as admission from other care providers are thus important variables predicting cost. Our study found these variables to be important predictors for high deficit. Although deficit and costs are not directly comparable, our findings are nevertheless not contradicted but rather supported by the published literature. Socioeconomic factors such as martial status, ethnicity and income or wealth also seem to be important. However, these factors were not captured by our dataset.

One of the main drivers of high resource utilization is length of stay. This is reflected in our results, 29.7% of deficit outliers being long stay outliers in comparison to 2.3% in the non-outlier cohort and 1.3% in the profit outlier group. However, when looking for additional predictors influencing outlier status, it would be problematic to include the predictor LOS into our analysis, as its inclusion would hide other correlated predicted effects of interest in all but the univariate analysis. As the DRG reimbursement system is supposed to break with the passed reimbursement system of daily premiums, we were precisely interested in finding these aforementioned effects of interest which would be helpful in making the reimbursement of outliers in the DRG-based reimbursement system more accurate, without resorting to daily premiums. If this can be achieved for all types of inpatient medical services remains to be seen. Furthermore, LOS is not just a causal agent of higher costs, but due to the Swiss cost accounting method of REKOLE® [4], a majority of costs are actually allocated to the cases according to the length of stay.

So far, PCCL score, amount of transfused RBC concentrates, dementia, psychiatric diagnoses, burns and osteoporosis directly or indirectly influence the reimbursement rate. However, a binary coding for ICU stay, mechanical ventilation, emergency admission and admission from another care provider do not. Nonetheless, other ICU scores already strongly influence the reimbursement rate, such as the number of hours of mechanical respiratory assistance. Emergency admission and admission from another care provider were probably not considered by the Swiss DRG institute, because of fears of lack of reliability on a national level. As the present Swiss DRG reimbursement system is not accurate enough to depict outliers. Therefore, we would welcome an assessment for an involvement of the aforementioned variables or their re-weighting in the DRG algorithm.

In its year of introduction in 2012, the Swiss DRG system led to balanced payments of non-outliers. However, roughly 10% of cases showed an average deficit of over 30,000 CHF and roughly 7% of cases an average profit of over 26,000 CHF. At both ends, the reimbursement system did not seem balanced. Since then, efforts have been undertaken to rebalance the system, either through an expansion of the supplemental payments, including expensive medications such as antifungal drugs and biologicals, or through a re-shifting of case weights, as is supposed to be the case for pediatric care in 2016. However, there has not been an effective and systematic approach of reducing the financial imbalances of outlier cases. [30].

We chose two methodically different variable selection methods in addition to multivariate regression and compared the accuracy of all three models with receiver operating characteristic curves. We selected a low threshold for significance (p<0.001). With 28,892 included cases, the study population was large. However, the data was collected from one year and one center only.

As a conclusion, we suggest considering psychiatric diagnosis, admission as an emergency case and admission from an external health care provider as DRG split criteria as they predict large, consistent and significant losses in earnings throughout all quantiles in the quantile regression analysis. Osteoporois may be useful in discerning cases with a predicted higher use of resources within certain DRGs.

## Supporting Information

S1 Appendix Univartiate and multivariate logistic regressions predicting deficit outliers (vs. non-outliers) and profit outliers (vs. non-outliers), IQR method. (DOCX)

S2 Appendix Analysis results–Model with outlier selection based on a 40% deviation of the DRG-amount to total costs in relation to total costs. (DOCX)

S1 Fig Outlier cases defined for 40% deviation (n = 28,892, test set). (TIF)

S2 Fig ROC-curves for the multivariate regression model and the two variable selection methods for the prediction of deficit outliers (outlier selection with 40% deviation) (n = 8,892, test set). (TIF)

S3 Fig ROC-curves for multivariate regression model and the two variable selection methods for the prediction of profit outliers (outlier selection with 40% deviation) (n = 8,892, test set). (TIF)
