# Supplementary material for: Predictors of High Profit and High Deficit Outliers under SwissDRG of a Tertiary Care Center
Source: PLoS One. 2015 Oct 30;10(10):e0140874. doi: 10.1371/journal.pone.0140874 (PMC4627843; doi:10.1371/journal.pone.0140874)
Supplement: S1 Appendix — (DOCX) [file pone.0140874.s001.docx]

**APPENDIX 1**

**Univartiate and multivariate logistic regressions predicting deficit outliers (vs. non-outliers) and profit outliers (vs. non-outliers).**

**Appendix 1 Table A** Univariate logistic regression predicting deficit outliers (vs. non-outliers). Outliers were defined by the IQR method. Results were expressed as odds ratio and p value.

| **Predictors** | **Odds ratio** | **p value** |
| --- | --- | --- |
| Acute renal insufficiency | 17.45 | < 0.0001 |
| Liver rupture | 16.87 | 0.0011 |
| Colon injury | 16.86 | 0.02 |
| Fracture of the acetabulum | 15.52 | < 0.0001 |
| Mechanical ventilation (binary yes-no) | 15.48 | < 0.0001 |
| SIRS | 14.95 | < 0.0001 |
| Hemothorax | 14.27 | < 0.0001 |
| Sepsis | 12.45 | < 0.0001 |
| Wound dehisence | 11.56 | < 0.0001 |
| ICU stay (binary yes-no) | 11.5 | < 0.0001 |
| Supplementary payments ("Zusatzentgelte" - binary yes-no) | 10.43 | < 0.0001 |
| Withdrawl syndrome with delirium | 9.78 | < 0.0001 |
| Epidural hematoma | 9.78 | < 0.0001 |
| Burns | 8.9 | < 0.0001 |
| Deep vein thrombosis | 8.55 | < 0.0001 |
| Rupture of the spleen | 8.44 | 0.0008 |
| Injury of the small intestine | 8.44 | 0.0026 |
| Right cardiac failure | 7.65 | < 0.0001 |
| Pneumonia | 7.55 | < 0.0001 |
| Fracture of the sacrum | 7.48 | < 0.0001 |
| Fracture of the calcaneus | 7.16 | < 0.0001 |
| Fracture of the scapula | 6.57 | 0.0002 |
| Postoperative wound infection | 6.55 | < 0.0001 |
| Respiratory insufficiency | 6.48 | < 0.0001 |
| Intracerebral bleeding excluding contusions | 6.2 | < 0.0001 |
| Subdural hematoma | 6.05 | < 0.0001 |
| Traumatic pneumothorax | 5.87 | < 0.0001 |
| Osteoporotic fracture | 5.68 | < 0.0001 |
| Complications of wound treatment | 5.63 | < 0.0001 |
| Pneumothorax (all diagnoses) | 5.47 | < 0.0001 |
| Hematoma or seroma | 5.45 | < 0.0001 |
| Hemiplegia | 5.25 | < 0.0001 |
| Plegia (all diagnoses) | 5.16 | < 0.0001 |
| Pulmonary injury | 5.15 | < 0.0001 |
| Intracerebral bleeding including contusions | 5.09 | < 0.0001 |
| Fracture of the cervical spine | 5 | < 0.0001 |
| Pulmonary embolism | 4.94 | < 0.0001 |
| Subarachnoidal bleeding | 4.92 | < 0.0001 |
| Sternum fracture | 4.69 | 0.0057 |
| Fracture of the thoracic spine | 4.48 | < 0.0001 |
| Cerebral infarction | 4.4 | < 0.0001 |
| Fracture of the lumbal spine | 4.35 | < 0.0001 |
| Admission from another care provider | 4.23 | < 0.0001 |
| Rupture of the bladder | 4.21 | 0.24 |
| Alcohol intoxication | 4.21 | 0.24 |
| Cardiac insufficiency | 4.14 | < 0.0001 |
| Instable thoracic cage / serial rip fracture | 4.09 | < 0.0001 |
| Cardiac arrythmia | 4.04 | < 0.0001 |
| Dementia | 3.96 | < 0.0001 |
| Fracture of the foot | 3.87 | < 0.0001 |
| Leukemia | 3.85 | < 0.0001 |
| Atrial fibrillation or flutter | 3.79 | < 0.0001 |
| Reoperation | 3.6 | < 0.0001 |
| Left cardiac insufficiency | 3.59 | < 0.0001 |
| Psychiatric diagnosis | 3.57 | < 0.0001 |
| Rib fracture | 3.43 | < 0.0001 |
| Osteoporosis | 3.22 | < 0.0001 |
| Chronic pulmonary illness | 3.2 | < 0.0001 |
| Chronic renal insufficiency (stage III and higher) | 3.13 | < 0.0001 |
| Fracture of the proximal humerus | 3.1 | 0.0014 |
| Pertrochantic fracture of the femur | 2.81 | 0.02 |
| Referral from our care hospital to another inpatient care proider | 2.7 | < 0.0001 |
| Fracture of the neurocranium | 2.5 | < 0.0001 |
| Arterial hypertension | 2.27 | < 0.0001 |
| Chronic alcoholic disease | 2.26 | < 0.0001 |
| Diabetes mellitus | 2.22 | < 0.0001 |
| Skull fracture (all types) | 2.19 | < 0.0001 |
| Depression | 2.17 | < 0.0001 |
| PCCL score | 2.16 | < 0.0001 |
| Neoplasm, malignant or of unknown malignancy | 2.11 | < 0.0001 |
| Fracture of the hand | 2.11 | 0.05 |
| Number of visits to the operating theatre | 2.03 | < 0.0001 |
| Malignant neoplasm | 2.02 | < 0.0001 |
| Adipositas | 1.9 | < 0.0001 |
| Fracture of the femoral neck | 1.87 | 0.16 |
| Acute myocardial infarction | 1.76 | 0.0001 |
| Fracture of the clavicula | 1.72 | 0.1 |
| LOS at the ICU (in days) | 1.7 | < 0.0001 |
| RBC concentrates | 1.69 | < 0.0001 |
| Luxation of the ellbow | 1.68 | 0.63 |
| Emergency admission | 1.68 | < 0.0001 |
| Thyroid disease | 1.67 | < 0.0001 |
| Coronary artery disease | 1.66 | < 0.0001 |
| Lymphoma or plasmocytoma | 1.65 | 0.0012 |
| Fracture of the malleolus | 1.48 | 0.25 |
| HIV | 1.47 | 0.05 |
| Peripheral arteriosclerosis | 1.45 | 0.001 |
| Dyslipedemia | 1.43 | < 0.0001 |
| Male sex | 1.35 | < 0.0001 |
| Fracture of the distal radius | 1.05 | 0.87 |
| Length of mechanical ventilation in h | 1.02 | < 0.0001 |
| Age | 1.02 | < 0.0001 |
| Intoxication with psychotropic substances | 0.96 | 0.91 |
| Commotio cerebri | 0.87 | 0.41 |
| Luxation of the shoulder | 0.00003 | 0.92 |

**Appendix 1 Table B** Results of Multivariate logistic regression predicting high deﬁcit. Outliers were selected with the IQR method. Results are given as odds ratio and p value.

| **Predictors** | **Odds ratio** | **p value** |
| --- | --- | --- |
| Alcohol intoxication | 12.81 | 0.08 |
| Burns | 5.42 | < 0.0001 |
| Fracture of the calcaneus | 4.28 | 0.08 |
| Liver rupture | 4.00 | 0.21 |
| Fracture of the acetabulum | 3.85 | 0.05 |
| Luxation of the ellbow | 3.00 | 0.39 |
| Epidural hematoma | 2.94 | 0.04 |
| ICU stay (binary yes-no) | 2.57 | < 0.0001 |
| Skull fracture (all types) | 2.50 | 0.0053 |
| Osteoporotic fracture | 2.39 | 0.0081 |
| Dementia | 2.36 | < 0.0001 |
| Respiratory insufficiency | 2.31 | < 0.0001 |
| Withdrawl syndrome with delirium | 2.24 | 0.13 |
| Cerebral infarction | 2.10 | < 0.0001 |
| Wound dehisence | 2.06 | 0.06 |
| Admission from another care provider | 1.97 | < 0.0001 |
| Fracture of the malleolus | 1.94 | 0.15 |
| Acute renal insufficiency | 1.92 | 0.0011 |
| Osteoporosis | 1.89 | < 0.0001 |
| Plegia (all diagnoses) | 1.76 | 0.02 |
| Fracture of the foot | 1.74 | 0.39 |
| Instable thoracic cage / serial rip fracture | 1.71 | 0.49 |
| Deep vein thrombosis | 1.71 | 0.15 |
| Subdural hematoma | 1.68 | 0.12 |
| Psychiatric diagnosis | 1.68 | < 0.0001 |
| Cardiac arrythmia | 1.67 | 0.0064 |
| Number of visits to the operating theatre | 1.61 | < 0.0001 |
| Fracture of the cervical spine | 1.57 | 0.35 |
| Reoperation | 1.55 | 0.0005 |
| PCCL score (score range 0.0 – 4.0) | 1.53 | < 0.0001 |
| Right cardiac failure | 1.52 | 0.16 |
| Fracture of the scapula | 1.52 | 0.63 |
| Intracerebral bleeding including contusions | 1.49 | 0.37 |
| Fracture of the proximal humerus | 1.49 | 0.47 |
| Emergency admission | 1.48 | < 0.0001 |
| Malignant neoplasm | 1.46 | 0.13 |
| Hematoma or seroma | 1.44 | 0.24 |
| Adipositas | 1.38 | 0.02 |
| Pneumonia | 1.37 | 0.03 |
| SIRS | 1.36 | 0.27 |
| Fracture of the hand | 1.33 | 0.60 |
| Pulmonary embolism | 1.32 | 0.35 |
| Fracture of the thoracic spine | 1.32 | 0.57 |
| Postoperative wound infection | 1.28 | 0.43 |
| Sternum fracture | 1.28 | 0.8 |
| Fracture of the sacrum | 1.28 | 0.66 |
| Subarachnoidal bleeding | 1.27 | 0.58 |
| Fracture of the distal radius | 1.26 | 0.56 |
| Pulmonary injury | 1.26 | 0.70 |
| Leukemia | 1.20 | 0.47 |
| RBC concentrates (number of transfused units) | 1.18 | < 0.0001 |
| Neoplasm, malignant or of unknown malignancy | 1.17 | 0.52 |
| Cardiac insufficiency | 1.17 | 0.71 |
| Lymphoma or plasmocytoma | 1.15 | 0.46 |
| Diabetes mellitus | 1.15 | 0.14 |
| Thyroid disease | 1.14 | 0.31 |
| Mechanical ventilation (binary yes-no) | 1.13 | 0.38 |
| Chronic pulmonary illness | 1.11 | 0.35 |
| Chronic renal insufficiency (stage III and higher) | 1.10 | 0.38 |
| Arterial hypertension | 1.08 | 0.33 |
| Sepsis | 1.08 | 0.82 |
| Injury of the small intestine | 1.06 | 0.96 |
| Depression | 1.06 | 0.70 |
| Intracerebral bleeding excluding contusions | 1.06 | 0.93 |
| LOS at the ICU (in days) | 1.05 | 0.03 |
| Peripheral arteriosclerosis | 1.02 | 0.88 |
| Male sex | 1.01 | 0.86 |
| Length of mechanical ventilation in h | 1.0 | 0.54 |
| Age (in years) | 1.0 | 0.03 |
| Pneumothorax (all diagnoses) | 0.97 | 0.95 |
| Fracture of the clavicula | 0.94 | 0.91 |
| Hemiplegia | 0.92 | 0.75 |
| Referral from our care hospital to another inpatient care proider | 0.88 | 0.27 |
| Coronary artery disease | 0.88 | 0.28 |
| Left cardiac insufficiency | 0.85 | 0.66 |
| Fracture of the lumbal spine | 0.85 | 0.74 |
| Dyslipedemia | 0.84 | 0.15 |
| HIV | 0.84 | 0.46 |
| Complications of wound treatment | 0.83 | 0.54 |
| Commotio cerebri | 0.82 | 0.38 |
| Chronic alcoholic disease | 0.78 | 0.25 |
| Pertrochantic fracture of the femur | 0.72 | 0.60 |
| Atrial fibrillation or flutter | 0.67 | 0.05 |
| Supplementary payments ("Zusatzentgelte" - binary yes-no) | 0.64 | 0.06 |
| Hemothorax | 0.59 | 0.29 |
| Rupture of the spleen | 0.59 | 0.59 |
| Traumatic pneumothorax | 0.57 | 0.49 |
| Fracture of the neurocranium | 0.50 | 0.08 |
| Acute myocardial infarction | 0.50 | 0.0012 |
| Intoxication with psychotropic substances | 0.48 | 0.07 |
| Fracture of the femoral neck | 0.43 | 0.14 |
| Rib fracture | 0.39 | 0.21 |
| Colon injury | 0.06 | 0.78 |
| Rupture of the bladder | 0.00 | 0.91 |
| Luxation of the shoulder | 0.00 | 0.94 |

**Appendix 1 Table C** Univariate logistic regression predicting profit outliers (vs. non-outliers). Outliers were defined by the IQR method. Results were expressed as odds ratio and 95% confidence interval.

| **Predictors** | **Odds ratio** | **p value** |
| --- | --- | --- |
| Burns | 21.8 | < 0.0001 |
| Leukemia | 10.38 | < 0.0001 |
| Mechanical ventilation (binary yes-no) | 10.03 | < 0.0001 |
| Fracture of the acetabulum | 7.8 | 0.0015 |
| Fracture of the sacrum | 7.6 | < 0.0001 |
| Supplementary payments ("Zusatzentgelte" - binary yes-no) | 7.25 | < 0.0001 |
| Sepsis | 6.99 | < 0.0001 |
| SIRS | 6.96 | < 0.0001 |
| ICU stay (binary yes-no) | 6.81 | < 0.0001 |
| Pulmonary injury | 6.13 | < 0.0001 |
| Acute renal insufficiency | 5.38 | < 0.0001 |
| sternum | 5.2 | 0.0061 |
| Respiratory insufficiency | 4.92 | < 0.0001 |
| Wound dehisence | 4.7 | < 0.0001 |
| Cardiac insufficiency | 4.36 | < 0.0001 |
| Left cardiac insufficiency | 4.26 | < 0.0001 |
| Hemothorax | 3.9 | 0.0018 |
| Postoperative wound infection | 3.49 | < 0.0001 |
| Hematoma or seroma | 3.37 | < 0.0001 |
| Complications of wound treatment | 3.36 | < 0.0001 |
| Right cardiac failure | 3.25 | < 0.0001 |
| Chronic renal insufficiency (stage III and higher) | 3.18 | < 0.0001 |
| Traumatic pneumothorax | 3.15 | 0.0071 |
| Instable thoracic cage / serial rip fracture | 3.06 | < 0.0001 |
| Lymphoma or plasmocytoma | 2.97 | < 0.0001 |
| Fracture of the thoracic spine | 2.93 | 0.004 |
| Injury of the small intestine | 2.92 | 0.34 |
| Pneumonia | 2.87 | < 0.0001 |
| Epidural hematoma | 2.7 | 0.12 |
| Rib fracture | 2.55 | 0.0001 |
| Neoplasm, malignant or of unknown malignancy | 2.51 | < 0.0001 |
| Malignant neoplasm | 2.51 | < 0.0001 |
| Pneumothorax (all diagnoses) | 2.5 | 0.0028 |
| Fracture of the lumbal spine | 2.45 | 0.01 |
| Cardiac arrythmia | 2.42 | < 0.0001 |
| Atrial fibrillation or flutter | 2.34 | < 0.0001 |
| Subdural hematoma | 2.31 | 0.0084 |
| Subarachnoidal bleeding | 2.25 | 0.02 |
| Acute myocardial infarction | 2.25 | < 0.0001 |
| Coronary artery disease | 2.16 | < 0.0001 |
| Fracture of the cervical spine | 2.12 | 0.17 |
| Reoperation | 2.03 | < 0.0001 |
| Arterial hypertension | 2 | < 0.0001 |
| Chronic pulmonary illness | 1.97 | < 0.0001 |
| Intracerebral bleeding including contusions | 1.85 | 0.09 |
| Dyslipedemia | 1.84 | < 0.0001 |
| Osteoporosis | 1.84 | < 0.0001 |
| Plegia (all diagnoses) | 1.79 | 0.0001 |
| PCCL score | 1.79 | < 0.0001 |
| Fracture of the clavicula | 1.73 | 0.15 |
| Male sex | 1.69 | < 0.0001 |
| Diabetes mellitus | 1.67 | < 0.0001 |
| Hemiplegia | 1.61 | 0.01 |
| Psychiatric diagnosis | 1.57 | < 0.0001 |
| Admission from another care provider | 1.56 | < 0.0001 |
| Number of visits to the operating theatre | 1.55 | < 0.0001 |
| Adipositas | 1.52 | 0.0008 |
| Pulmonary embolism | 1.44 | 0.37 |
| Deep vein thrombosis | 1.42 | 0.51 |
| RBC concentrates | 1.4 | < 0.0001 |
| Peripheral arteriosclerosis | 1.38 | 0.01 |
| LOS at the ICU (in days) | 1.32 | < 0.0001 |
| Fracture of the femoral neck | 1.3 | 0.67 |
| Referral from our care hospital to another inpatient care proider | 1.26 | 0.06 |
| Osteoporotic fracture | 1.2 | 0.73 |
| Depression | 1.15 | 0.32 |
| Pertrochantic fracture of the femur | 1.11 | 0.89 |
| HIV | 1.08 | 0.77 |
| Thyroid disease | 1.07 | 0.66 |
| Age | 1.02 | < 0.0001 |
| Length of mechanical ventilation in h | 1.01 | < 0.0001 |
| Fracture of the neurocranium | 1.01 | 0.97 |
| Fracture of the foot | 1 | 1 |
| Fracture of the calcaneus | 0.9 | 0.92 |
| Withdrawl syndrome with delirium | 0.9 | 0.92 |
| Chronic alcoholic disease | 0.9 | 0.68 |
| Fracture of the distal radius | 0.79 | 0.59 |
| Intracerebral bleeding excluding contusions | 0.78 | 0.81 |
| Cerebral infarction | 0.73 | 0.29 |
| Skull fracture (all types) | 0.71 | 0.15 |
| Dementia | 0.65 | 0.26 |
| Emergency admission | 0.6 | < 0.0001 |
| Commotio cerebri | 0.45 | 0.0024 |
| Fracture of the malleolus | 0.41 | 0.21 |
| Fracture of the proximal humerus | 0.39 | 0.35 |
| Fracture of the hand | 0.32 | 0.26 |
| Intoxication with psychotropic substances | 0.24 | 0.05 |
| Colon injury | 0.0003 | 0.95 |
| Alcohol intoxication | 0.0001 | 0.95 |
| Liver rupture | 0.0001 | 0.95 |
| Rupture of the bladder | 0.0001 | 0.95 |
| Rupture of the spleen | 0.00004 | 0.94 |
| Luxation of the ellbow | 0.00004 | 0.94 |
| Fracture of the scapula | 0.00001 | 0.95 |
| Luxation of the shoulder | 0.00001 | 0.95 |

**Appendix 1 Table D** Results of multivariate logistic regression predicting high profit. Outliers were selected with the IQR method. Results are given as odds ratio and p value.

| **Predictors** | **Odds ratio** | **P value** |
| --- | --- | --- |
| Fracture of the neurocranium | 1118681.4 | 0.95 |
| Burns | 62.04 | < 0.0001 |
| Leukemia | 6.35 | < 0.0001 |
| Cardiac insufficiency | 5.57 | 0.001 |
| Fracture of the acetabulum | 4.97 | 0.07 |
| Sternum fracture | 3.91 | 0.17 |
| ICU stay (binary yes-no) | 3.54 | < 0.0001 |
| Fracture of the sacrum | 3.40 | 0.03 |
| Instable thoracic cage / serial rip fracture | 2.67 | 0.30 |
| Pulmonary injury | 2.52 | 0.10 |
| Supplementary payments ("Zusatzentgelte" - binary yes-no) | 2.43 | < 0.0001 |
| Respiratory insufficiency | 2.29 | < 0.0001 |
| Fracture of the lumbal spine | 2.10 | 0.16 |
| Injury of the small intestine | 1.95 | 0.58 |
| Subarachnoidal bleeding | 1.93 | 0.27 |
| Lymphoma or plasmocytoma | 1.85 | 0.0002 |
| Fracture of the foot | 1.8 | 0.50 |
| Sepsis | 1.71 | 0.18 |
| Intracerebral bleeding including contusions | 1.70 | 0.39 |
| Fracture of the cervical spine | 1.68 | 0.42 |
| Wound dehisence | 1.55 | 0.32 |
| PCCL score (score range 0.0 – 4.0) | 1.54 | < 0.0001 |
| Plegia (all diagnoses) | 1.53 | 0.16 |
| Acute myocardial infarction | 1.45 | 0.07 |
| Subdural hematoma | 1.42 | 0.49 |
| SIRS | 1.41 | 0.35 |
| Hematoma or seroma | 1.38 | 0.34 |
| Neoplasm, malignant or of unknown malignancy | 1.36 | 0.28 |
| Pneumothorax (all diagnoses) | 1.35 | 0.61 |
| Cardiac arrythmia | 1.35 | 0.16 |
| Mechanical ventilation (binary yes-no) | 1.33 | 0.05 |
| Number of visits to the operating theatre | 1.32 | < 0.0001 |
| Traumatic pneumothorax | 1.31 | 0.77 |
| Postoperative wound infection | 1.31 | 0.43 |
| Epidural hematoma | 1.30 | 0.77 |
| Male sex | 1.29 | 0.0001 |
| Psychiatric diagnosis | 1.24 | 0.05 |
| Osteoporosis | 1.24 | 0.23 |
| Reoperation | 1.22 | 0.14 |
| Dyslipedemia | 1.16 | 0.19 |
| Chronic renal insufficiency (stage III and higher) | 1.16 | 0.22 |
| Arterial hypertension | 1.14 | 0.10 |
| Fracture of the clavicula | 1.12 | 0.85 |
| Malignant neoplasm | 1.10 | 0.74 |
| Fracture of the distal radius | 1.06 | 0.91 |
| Coronary artery disease | 1.05 | 0.68 |
| Deep vein thrombosis | 1.04 | 0.95 |
| RBC concentrates (number of transfused units) | 1.01 | 0.64 |
| Length of mechanical ventilation in h | 1.01 | < 0.0001 |
| Age (in years) | 1.00 | 0.03 |
| Pertrochantic fracture of the femur | 0.96 | 0.96 |
| Adipositas | 0.96 | 0.76 |
| Fracture of the thoracic spine | 0.95 | 0.93 |
| Depression | 0.92 | 0.67 |
| Complications of wound treatment | 0.92 | 0.80 |
| LOS at the ICU (in days) | 0.91 | 0.0008 |
| Commotio cerebri | 0.89 | 0.74 |
| Peripheral arteriosclerosis | 0.88 | 0.41 |
| Diabetes mellitus | 0.85 | 0.12 |
| Thyroid disease | 0.85 | 0.30 |
| Chronic pulmonary illness | 0.84 | 0.18 |
| HIV | 0.83 | 0.51 |
| Fracture of the malleolus | 0.76 | 0.73 |
| Admission from another care provider | 0.76 | 0.03 |
| Acute renal insufficiency | 0.72 | 0.25 |
| Fracture of the femoral neck | 0.71 | 0.63 |
| Chronic alcoholic disease | 0.71 | 0.25 |
| Fracture of the calcaneus | 0.70 | 0.80 |
| Fracture of the proximal humerus | 0.70 | 0.73 |
| Pulmonary embolism | 0.65 | 0.37 |
| Pneumonia | 0.64 | 0.03 |
| Withdrawl syndrome with delirium | 0.63 | 0.71 |
| Referral from our care hospital to another inpatient care proider | 0.63 | 0.0052 |
| Atrial fibrillation or flutter | 0.62 | 0.04 |
| Osteoporotic fracture | 0.60 | 0.41 |
| Cerebral infarction | 0.60 | 0.14 |
| Hemiplegia | 0.54 | 0.09 |
| Emergency admission | 0.47 | < 0.0001 |
| Rib fracture | 0.46 | 0.38 |
| Dementia | 0.44 | 0.06 |
| Right cardiac failure | 0.38 | 0.02 |
| Left cardiac insufficiency | 0.38 | 0.05 |
| Hemothorax | 0.30 | 0.08 |
| Intoxication with psychotropic substances | 0.21 | 0.05 |
| Fracture of the hand | 0.12 | 0.13 |
| Intracerebral bleeding excluding contusions | 0.09 | 0.06 |
| Alcohol intoxication | 0.00 | 0.99 |
| Colon injury | 0.00 | 1.00 |
| Liver rupture | 0.00 | 0.99 |
| Rupture of the bladder | 0.00 | 0.99 |
| Luxation of the ellbow | 0.00 | 0.99 |
| Skull fracture (all types) | 0.00 | 0.95 |
| Fracture of the scapula | 0.00 | 0.98 |
| Rupture of the spleen | 0.000 | 0.99 |
| Luxation of the shoulder | 0.00 | 0.98 |
