# Supplementary material for: Predictors of High Profit and High Deficit Outliers under SwissDRG of a Tertiary Care Center
Source: PLoS One. 2015 Oct 30;10(10):e0140874. doi: 10.1371/journal.pone.0140874 (PMC4627843; doi:10.1371/journal.pone.0140874)
Supplement: S2 Appendix — (DOCX) [file pone.0140874.s002.docx]

**APPENDIX 2**

**Analysis results – Model with outlier selection based on a 40% deviation of the DRG-amount to total costs in relation to total costs**

**Appendix 2 Table A** Sample characteristics. Outliers selected with the % deviation method.

|  | **All cases** | **Non-outliers** | **Deficit outliers** | **Profit outliers** |
| --- | --- | --- | --- | --- |
| **Number of cases** | **n=28‘892** | **n=17‘614** | **n=3‘217** | **n=8‘061** |
| **Age (in years)** | 46.5 (23.9) | 46.9 (23.1) | 50.9 (23.1) | 43.8 (25.8) |
| **Sex (female in %)** | 50.2% | 51.9% | 46.9% | 47.8% |
| **Mortality (%)** | 2.3% | 1.9% | 7.2% | 1.2% |
| **Emergency admission (%)** | 40.9% | 41.2% | 57.6% | 33.7% |
| **Mechanical ventilation (%)** | 6.5% | 6.9% | 13.4% | 2.9% |
| **Length of mechanical ventilation in h (mean and SD)** | 7.5 (61.8) | 8.7 (67.7) | 13.3 (79.8) | 2.7 (32.9) |
| **LOS at ICU in days (mean and SD)** | 0.5 (3.5) | 0.6 (3.8) | 1.2 (5.2) | 0.1 (0.7) |
| **Admission from other care providers** | 7.4% | 7.1% | 16.1% | 4.6% |

**Appendix 2 Table B** Univariate logistic regression predicting deficit outliers (vs. non-outliers). Outliers were defined by the % deviation method. Results were expressed as odds ratio and p value.

| **Predictors** | **Odds ratio** | **p value** |
| --- | --- | --- |
| Withdrawl syndrome with delirium | 7.52 | < 0.0001 |
| Fracture of the acetabulum | 5.5 | 0.0007 |
| Intracerebral bleeding excluding contusions | 4.66 | 0.0002 |
| Dementia | 4.1 | < 0.0001 |
| Fracture of the foot | 3.83 | < 0.0001 |
| Fracture of the cervical spine | 3.58 | 0.0004 |
| Osteoporotic fracture | 3.53 | < 0.0001 |
| Fracture of the scapula | 3.3 | 0.02 |
| Injury of the small intestine | 3.29 | 0.1 |
| Deep vein thrombosis | 3.28 | < 0.0001 |
| Traumatic pneumothorax | 3.08 | 0.0008 |
| Subdural hematoma | 3.03 | < 0.0001 |
| Intoxication with psychotropic substances | 2.98 | < 0.0001 |
| Psychiatric diagnosis | 2.92 | < 0.0001 |
| Hemothorax | 2.9 | 0.0001 |
| Fracture of the sacrum | 2.89 | 0.0066 |
| Pulmonary injury | 2.87 | 0.0031 |
| Burns | 2.86 | 0.0021 |
| Acute renal insufficiency | 2.85 | < 0.0001 |
| Fracture of the lumbal spine | 2.83 | 0.0002 |
| Plegia (all diagnoses) | 2.8 | < 0.0001 |
| Right cardiac failure | 2.79 | < 0.0001 |
| Subarachnoidal bleeding | 2.71 | < 0.0001 |
| Hemiplegia | 2.69 | < 0.0001 |
| Sepsis | 2.66 | < 0.0001 |
| Fracture of the thoracic spine | 2.59 | 0.0017 |
| Admission from another care provider | 2.58 | < 0.0001 |
| Chronic alcoholic disease | 2.56 | < 0.0001 |
| Intracerebral bleeding including contusions | 2.56 | 0.0001 |
| SIRS | 2.54 | < 0.0001 |
| Pulmonary embolism | 2.51 | 0.0002 |
| Pneumonia | 2.46 | < 0.0001 |
| Respiratory insufficiency | 2.38 | < 0.0001 |
| Pneumothorax (all diagnoses) | 2.34 | 0.0004 |
| Depression | 2.31 | < 0.0001 |
| Lymphoma or plasmocytoma | 2.28 | < 0.0001 |
| Cerebral infarction | 2.26 | < 0.0001 |
| ICU stay (binary yes-no) | 2.21 | < 0.0001 |
| Fracture of the hand | 2.2 | 0.02 |
| Osteoporosis | 2.2 | < 0.0001 |
| leukemia | 2.17 | 0.0002 |
| Fracture of the malleolus | 2.16 | 0.03 |
| Referral from our care hospital to another inpatient care proider | 2.14 | < 0.0001 |
| Instable thoracic cage / serial rip fracture | 2.1 | 0.0011 |
| Mechanical ventilation (binary yes-no) | 2.05 | < 0.0001 |
| Rib fracture | 2.03 | 0.0006 |
| Emergency admission | 1.99 | < 0.0001 |
| Fracture of the proximal humerus | 1.98 | 0.08 |
| Fracture of the neurocranium | 1.87 | < 0.0001 |
| Luxation of the ellbow | 1.83 | 0.6 |
| Skull fracture (all types) | 1.8 | < 0.0001 |
| Cardiac arrythmia | 1.77 | < 0.0001 |
| Atrial fibrillation or flutter | 1.75 | < 0.0001 |
| Cardiac insufficiency | 1.74 | < 0.0001 |
| Wound dehisence | 1.74 | 0.04 |
| Epidural hematoma | 1.73 | 0.24 |
| Hematoma or seroma | 1.69 | < 0.0001 |
| Chronic renal insufficiency (stage III and higher) | 1.66 | < 0.0001 |
| Supplementary payments ("Zusatzentgelte" - binary yes-no) | 1.66 | 0.0005 |
| Chronic pulmonary illness | 1.62 | < 0.0001 |
| Reoperation | 1.51 | < 0.0001 |
| sternum | 1.5 | 0.54 |
| Left cardiac insufficiency | 1.49 | 0.0012 |
| Fracture of the clavicula | 1.47 | 0.25 |
| Diabetes mellitus | 1.47 | < 0.0001 |
| Complications of wound treatment | 1.47 | 0.0002 |
| Neopplasm, malignant or of unknown malignancy | 1.46 | < 0.0001 |
| Malignant neoplasm | 1.45 | < 0.0001 |
| Fracture of the femoral neck | 1.43 | 0.43 |
| Thyroid disease | 1.38 | 0.0027 |
| PCCL score | 1.36 | < 0.0001 |
| Arterial hypertension | 1.32 | < 0.0001 |
| Postoperative wound infection | 1.3 | 0.16 |
| HIV | 1.27 | 0.26 |
| Commotio cerebri | 1.26 | 0.08 |
| Male sex | 1.25 | < 0.0001 |
| Fracture of the distal radius | 1.21 | 0.47 |
| Adipositas | 1.11 | 0.34 |
| RBC concentrates | 1.05 | < 0.0001 |
| LOS at the ICU (in days) | 1.04 | < 0.0001 |
| Number of visits to the operating theatre | 1.01 | 0.76 |
| Age | 1.01 | < 0.0001 |
| Length of mechanical ventilation in h | 1 | 0.0004 |
| Peripheral arteriosclerosis | 0.97 | 0.8 |
| Dyslipedemia | 0.91 | 0.28 |
| Pertrochantic fracture of the femur | 0.75 | 0.64 |
| Acute myocardial infarction | 0.68 | 0.04 |
| Coronary artery disease | 0.65 | < 0.0001 |

**Appendix 2 Table C** 10 most important predictors for deﬁcit outliers defined by the % deviation method derived from Random Forest analysis.

| **Predictors** | **Accuracy** |
| --- | --- |
| Psychiatric diagnosis | 28.89 |
| Number of visits to the operating theatre | 26.33 |
| LOS at the ICU in days | 25.74 |
| RBC concentrates (number of transfused units) | 25.57 |
| Age (in years) | 23.67 |
| PCCL score (score range 0.0 - 4.0) | 19.18 |
| Coronary artery disease | 18.35 |
| Length of mechanical ventilation in h | 15.83 |
| Admission from another care provider | 15.82 |
| Emergency admission | 15.27 |

**Appendix 2 Table D** 10 most important predictors for deficit outliers defined by the % deviation method, derived from the L1 regularized logistic regression (Lasso). Predictors were ordered by the magnitude of their odds ratio.

| **Predictors** | **Odds ratio** |
| --- | --- |
| Psychiatric diagnosis | 1.92 |
| Admission from another care provider | 1.69 |
| Emergency admission | 1.48 |
| Lymphoma or plasmocytoma | 1.31 |
| PCCL score (score range 0.0 - 4.0) | 1.31 |
| Fracture of the foot | 1.31 |
| Dementia | 1.27 |
| Plegia (all diagnoses) | 1.21 |
| Respiratory insufficiency | 1.17 |
| Osteoporosis | 1.15 |

**Appendix 2 Table E** Results of multivariate logistic regression predicting high deﬁcit. Outliers were selected with the % deviation method. Results are given as odds ratio and p value.

| **Predictors** | **Odds ratio** | **P value** |
| --- | --- | --- |
| Injury of the small intestine | 3.72 | 0.10 |
| Fracture of the foot | 2.96 | 0.0026 |
| Intracerebral bleeding excluding contusions | 2.80 | 0.07 |
| Withdrawl syndrome with delirium | 2.79 | 0.02 |
| Fracture of the malleolus | 2.11 | 0.06 |
| Psychiatric diagnosis | 2.08 | < 0.0001 |
| Admission from another care provider | 2.05 | < 0.0001 |
| Fracture of the scapula | 1.99 | 0.31 |
| Fracture of the cervical spine | 1.93 | 0.10 |
| Fracture of the acetabulum | 1.91 | 0.27 |
| Dementia | 1.87 | 0.0006 |
| Lymphoma or plasmocytoma | 1.86 | 0.0002 |
| Fracture of the hand | 1.86 | 0.10 |
| Emergency admission | 1.76 | < 0.0001 |
| Skull fracture (all types) | 1.75 | 0.04 |
| Osteoporotic fracture | 1.74 | 0.07 |
| Plegia (all diagnoses) | 1.74 | 0.0096 |
| Respiratory insufficiency | 1.73 | < 0.0001 |
| Hematoma or seroma | 1.72 | 0.06 |
| Osteoporosis | 1.57 | 0.0011 |
| Fracture of the lumbal spine | 1.51 | 0.24 |
| Subdural hematoma | 1.49 | 0.18 |
| ICU stay (binary yes-no) | 1.48 | < 0.0001 |
| Deep vein thrombosis | 1.47 | 0.21 |
| Reoperation | 1.46 | 0.0006 |
| Luxation of the ellbow | 1.45 | 0.77 |
| Acute renal insufficiency | 1.41 | 0.05 |
| Cardiac insufficiency | 1.39 | 0.39 |
| Cardiac arrythmia | 1.37 | 0.05 |
| Fracture of the distal radius | 1.34 | 0.32 |
| Referral from our care hospital to another inpatient care proider | 1.29 | 0.0056 |
| Pulmonary injury | 1.29 | 0.58 |
| Intoxication with psychotropic substances | 1.27 | 0.3 |
| Malignant neoplasm | 1.26 | 0.33 |
| Traumatic pneumothorax | 1.24 | 0.72 |
| Sepsis | 1.23 | 0.44 |
| Wound dehisence | 1.22 | 0.57 |
| Thyroid disease | 1.20 | 0.12 |
| Male sex | 1.18 | 0.0014 |
| PCCL score (score range 0.0 - 4.0) | 1.18 | < 0.0001 |
| Right cardiac failure | 1.16 | 0.60 |
| Cerebral infarction | 1.16 | 0.38 |
| Diabetes mellitus | 1.15 | 0.10 |
| Pulmonary embolism | 1.15 | 0.62 |
| Depression | 1.12 | 0.33 |
| Fracture of the proximal humerus | 1.11 | 0.80 |
| Leukemia | 1.10 | 0.68 |
| Adipositas | 1.09 | 0.49 |
| Burns | 1.06 | 0.88 |
| Chronic renal insufficiency (stage III and higher) | 1.06 | 0.61 |
| Pneumonia | 1.05 | 0.69 |
| Instable thoracic cage / serial rip fracture | 1.05 | 0.94 |
| Subarachnoidal bleeding | 1.04 | 0.92 |
| RBC concentrates (number of transfused units) | 1.04 | < 0.0001 |
| Neoplasm, malignant or of unknown malignancy | 1.01 | 0.95 |
| Fracture of the thoracic spine | 1.01 | 0.97 |
| LOS at the ICU (in days) | 1.00 | 0.93 |
| Age (in years) | 1.00 | 0.04 |
| Length of mechanical ventilation in h | 1.00 | 0.0005 |
| Arterial hypertension | 0.96 | 0.57 |
| Chronic pulmonary illness | 0.96 | 0.68 |
| Number of visits to the operating theatre | 0.95 | 0.08 |
| Peripheral arteriosclerosis | 0.95 | 0.70 |
| Fracture of the sacrum | 0.93 | 0.88 |
| Fracture of the clavicula | 0.89 | 0.78 |
| Postoperative wound infection | 0.89 | 0.67 |
| Dyslipedemia | 0.87 | 0.2 |
| Commotio cerebri | 0.86 | 0.33 |
| SIRS | 0.86 | 0.53 |
| Pneumothorax (all diagnoses) | 0.84 | 0.65 |
| Hemothorax | 0.84 | 0.62 |
| Atrial fibrillation or flutter | 0.83 | 0.31 |
| Chronic alcoholic disease | 0.82 | 0.25 |
| Mechanical ventilation (binary yes-no) | 0.80 | 0.07 |
| Hemiplegia | 0.79 | 0.33 |
| Intracerebral bleeding including contusions | 0.78 | 0.56 |
| sternum | 0.77 | 0.74 |
| Epidural hematoma | 0.77 | 0.63 |
| Left cardiac insufficiency | 0.74 | 0.38 |
| HIV | 0.73 | 0.15 |
| Complications of wound treatment | 0.70 | 0.22 |
| Fracture of the femoral neck | 0.69 | 0.47 |
| Rib fracture | 0.66 | 0.47 |
| Fracture of the neurocranium | 0.62 | 0.14 |
| Coronary artery disease | 0.53 | < 0.0001 |
| Supplementary payments ("Zusatzentgelte" - binary yes-no) | 0.51 | 0.0014 |
| Acute myocardial infarction | 0.49 | 0.0009 |
| Pertrochantic fracture of the femur | 0.33 | 0.10 |

**Appendix 2 Table F** Univariate logistic regression predicting profit outliers (vs. non-outliers). Outliers were defined by the % deviation method. Results were expressed as odds ratio and p value.

| **Predictors** | **Odds ratio** | **p value** |
| --- | --- | --- |
| Burns | 4.09 | < 0.0001 |
| Leukemia | 2.39 | < 0.0001 |
| Fracture of the malleolus | 2.33 | 0.0013 |
| Lymphoma or plasmocytoma | 1.83 | < 0.0001 |
| Fracture of the sacrum | 1.6 | 0.18 |
| Luxation of the ellbow | 1.44 | 0.69 |
| Malignant neoplasm | 1.44 | < 0.0001 |
| Neopplasm, malignant or of unknown malignancy | 1.42 | < 0.0001 |
| Fracture of the foot | 1.41 | 0.3 |
| Fracture of the acetabulum | 1.35 | 0.6 |
| Chronic renal insufficiency (stage III and higher) | 1.34 | < 0.0001 |
| Pulmonary injury | 1.32 | 0.42 |
| Peripheral arteriosclerosis | 1.22 | 0.01 |
| Male sex | 1.15 | < 0.0001 |
| Fracture of the clavicula | 1.11 | 0.7 |
| HIV | 1.1 | 0.54 |
| Pneumothorax (all diagnoses) | 1.06 | 0.78 |
| Traumatic pneumothorax | 1.04 | 0.91 |
| Left cardiac insufficiency | 1.03 | 0.77 |
| Thyroid disease | 1.01 | 0.9 |
| Length of mechanical ventilation in h | 1 | < 0.0001 |
| PCCL score | 1 | 0.68 |
| Age | 1 | < 0.0001 |
| Cardiac insufficiency | 0.99 | 0.93 |
| Diabetes mellitus | 0.97 | 0.57 |
| Reoperation | 0.95 | 0.5 |
| RBC concentrates | 0.89 | < 0.0001 |
| Fracture of the thoracic spine | 0.89 | 0.72 |
| Instable thoracic cage / serial rip fracture | 0.88 | 0.57 |
| Arterial hypertension | 0.88 | 0.0012 |
| Postoperative wound infection | 0.88 | 0.39 |
| Osteoporosis | 0.87 | 0.21 |
| Dyslipedemia | 0.87 | 0.02 |
| Supplementary payments ("Zusatzentgelte" - binary yes-no) | 0.85 | 0.21 |
| Intracerebral bleeding including contusions | 0.84 | 0.5 |
| Fracture of the lumbal spine | 0.83 | 0.55 |
| Rib fracture | 0.82 | 0.32 |
| LOS at the ICU (in days) | 0.81 | < 0.0001 |
| Adipositas | 0.8 | 0.01 |
| Complications of wound treatment | 0.79 | 0.01 |
| Chronic pulmonary illness | 0.79 | 0.0024 |
| sternum | 0.79 | 0.68 |
| Acute myocardial infarction | 0.77 | 0.03 |
| Hematoma or seroma | 0.76 | 0.02 |
| Emergency admission | 0.74 | < 0.0001 |
| Chronic alcoholic disease | 0.74 | 0.06 |
| Respiratory insufficiency | 0.73 | 0.0023 |
| Fracture of the neurocranium | 0.73 | 0.03 |
| Sepsis | 0.73 | 0.03 |
| Atrial fibrillation or flutter | 0.71 | < 0.0001 |
| Coronary artery disease | 0.71 | < 0.0001 |
| Right cardiac failure | 0.7 | 0.06 |
| Pneumonia | 0.69 | 0.0027 |
| Fracture of the proximal humerus | 0.69 | 0.37 |
| Epidural hematoma | 0.68 | 0.42 |
| Subarachnoidal bleeding | 0.68 | 0.18 |
| Depression | 0.67 | < 0.0001 |
| Cardiac arrythmia | 0.67 | < 0.0001 |
| Pulmonary embolism | 0.67 | 0.15 |
| Plegia (all diagnoses) | 0.66 | 0.0003 |
| Fracture of the femoral neck | 0.66 | 0.33 |
| Psychiatric diagnosis | 0.66 | < 0.0001 |
| Skull fracture (all types) | 0.65 | 0.0011 |
| Fracture of the cervical spine | 0.65 | 0.35 |
| Deep vein thrombosis | 0.64 | 0.2 |
| SIRS | 0.63 | 0.0005 |
| Admission from another care provider | 0.62 | < 0.0001 |
| Wound dehisence | 0.61 | 0.07 |
| Osteoporotic fracture | 0.6 | 0.15 |
| Withdrawl syndrome with delirium | 0.59 | 0.42 |
| Number of visits to the operating theatre | 0.58 | < 0.0001 |
| Subdural hematoma | 0.57 | 0.04 |
| Dementia | 0.56 | 0.01 |
| Intracerebral bleeding excluding contusions | 0.5 | 0.28 |
| Pertrochantic fracture of the femur | 0.49 | 0.15 |
| Hemiplegia | 0.47 | < 0.0001 |
| ICU stay (binary yes-no) | 0.45 | < 0.0001 |
| Injury of the small intestine | 0.43 | 0.44 |
| Mechanical ventilation (binary yes-no) | 0.43 | < 0.0001 |
| Acute renal insufficiency | 0.41 | < 0.0001 |
| Referral from our care hospital to another inpatient care proider | 0.37 | < 0.0001 |
| Cerebral infarction | 0.31 | < 0.0001 |
| Fracture of the distal radius | 0.31 | 0.0003 |
| Intoxication with psychotropic substances | 0.3 | 0.0007 |
| Fracture of the hand | 0.29 | 0.02 |
| Hemothorax | 0.28 | 0.0082 |
| Commotio cerebri | 0.27 | < 0.0001 |
| Fracture of the scapula | 0.000008 | 0.91 |

**Appendix 2 Table G** 10 most important predictors for profit outliers defined by the % deviation method derived from Random Forest analysis.

| **Predictors** | **Accuracy** |
| --- | --- |
| Number of visits to the operating theatre | 58.1 |
| Burns | 36.66 |
| PCCL score | 33.6 |
| Referral from our care hospital to another inpatient care proider | 32.52 |
| Age | 27.78 |
| LOS at the ICU (in days) | 27.6 |
| Commotio cerebri | 27.27 |
| Neopplasm, malignant or of unknown malignancy | 26.31 |
| Admission from another care provider | 24.35 |
| RBC concentrates | 21.83 |

**Appendix 2 Table H** 10 most important predictors for deficit outliers defined by the % deviation method, derived from the L1 regularized logistic regression (Lasso). Predictors were ordered by the magnitude of their odds ratio.

| **Predictors** | **Odds_ratio** |
| --- | --- |
| Burns | 4.31 |
| Fracture of the malleolus | 2.28 |
| Fracture of the sacrum | 1.5 |
| Chronic renal insufficiency (stage III and higher) | 1.44 |
| Pulmonary injury | 1.42 |
| Neopplasm, malignant or of unknown malignancy | 1.24 |
| Leukemia | 1.21 |
| Fracture of the foot | 1.2 |
| Reoperation | 1.15 |
| Pneumothorax (all diagnoses) | 1.13 |

**Appendix 2 Table I** Results of multivariate logistic regression predicting high profit. Outliers were selected with the % deviation method. Results are given as odds ratio and p value.

| **Predictors** | **Odds ratio** | **p value** |
| --- | --- | --- |
| Burns | 8.76 | < 0.0001 |
| Fracture of the acetabulum | 3.8 | 0.06 |
| Fracture of the malleolus | 3.38 | < 0.0001 |
| Pulmonary injury | 2.19 | 0.1 |
| Instable thoracic cage / serial rip fracture | 2.15 | 0.21 |
| Fracture of the foot | 2.06 | 0.04 |
| Fracture of the sacrum | 1.87 | 0.17 |
| Cardiac insufficiency | 1.8 | 0.16 |
| Pneumothorax (all diagnoses) | 1.77 | 0.09 |
| Fracture of the neurocranium | 1.77 | 0.1 |
| Acute myocardial infarction | 1.68 | 0.0003 |
| leukemia | 1.65 | 0.004 |
| Luxation of the ellbow | 1.64 | 0.6 |
| Plegia (all diagnoses) | 1.61 | 0.02 |
| sternum | 1.61 | 0.51 |
| Withdrawl syndrome with delirium | 1.6 | 0.52 |
| Supplementary payments ("Zusatzentgelte" - binary yes-no) | 1.56 | 0.0082 |
| Chronic renal insufficiency (stage III and higher) | 1.56 | < 0.0001 |
| Fracture of the clavicula | 1.52 | 0.16 |
| Postoperative wound infection | 1.5 | 0.12 |
| Neopplasm, malignant or of unknown malignancy | 1.41 | 0.05 |
| Atrial fibrillation or flutter | 1.35 | 0.09 |
| Reoperation | 1.35 | 0.00032 |
| Intracerebral bleeding including contusions | 1.34 | 0.43 |
| SIRS | 1.21 | 0.54 |
| Fracture of the cervical spine | 1.2 | 0.73 |
| Male sex | 1.19 | < 0.0001 |
| Mechanical ventilation (binary yes-no) | 1.18 | 0.22 |
| Hematoma or seroma | 1.17 | 0.54 |
| Fracture of the proximal humerus | 1.17 | 0.71 |
| Peripheral arteriosclerosis | 1.12 | 0.21 |
| Thyroid disease | 1.11 | 0.23 |
| Fracture of the lumbal spine | 1.1 | 0.79 |
| Fracture of the thoracic spine | 1.09 | 0.82 |
| Fracture of the femoral neck | 1.09 | 0.85 |
| Chronic alcoholic disease | 1.09 | 0.65 |
| HIV | 1.07 | 0.69 |
| PCCL score | 1.04 | 0.0057 |
| Diabetes mellitus | 1.04 | 0.6 |
| Lymphoma or plasmocytoma | 1.01 | 0.96 |
| Malignant neoplasm | 1 | 1 |
| Length of mechanical ventilation in h | 1 | 0.38 |
| Age | 1 | 0.05 |
| Complications of wound treatment | 1 | 0.98 |
| Traumatic pneumothorax | 0.99 | 0.99 |
| Adipositas | 0.96 | 0.68 |
| Wound dehisence | 0.96 | 0.9 |
| Sepsis | 0.95 | 0.88 |
| Dyslipedemia | 0.94 | 0.45 |
| Arterial hypertension | 0.94 | 0.22 |
| Pneumonia | 0.94 | 0.64 |
| RBC concentrates | 0.92 | < 0.0001 |
| Subarachnoidal bleeding | 0.91 | 0.82 |
| Pulmonary embolism | 0.91 | 0.77 |
| LOS at the ICU (in days) | 0.9 | < 0.0001 |
| Chronic pulmonary illness | 0.89 | 0.18 |
| Pertrochantic fracture of the femur | 0.89 | 0.83 |
| Depression | 0.86 | 0.21 |
| Osteoporosis | 0.84 | 0.17 |
| Admission from another care provider | 0.83 | 0.03 |
| Left cardiac insufficiency | 0.8 | 0.59 |
| Psychiatric diagnosis | 0.8 | 0.0021 |
| ICU stay (binary yes-no) | 0.79 | 0.02 |
| Skull fracture (all types) | 0.79 | 0.43 |
| Epidural hematoma | 0.77 | 0.63 |
| Deep vein thrombosis | 0.76 | 0.47 |
| Acute renal insufficiency | 0.75 | 0.18 |
| Injury of the small intestine | 0.73 | 0.77 |
| Emergency admission | 0.71 | < 0.0001 |
| Right cardiac failure | 0.69 | 0.19 |
| Subdural hematoma | 0.67 | 0.27 |
| Respiratory insufficiency | 0.67 | 0.0015 |
| Rib fracture | 0.66 | 0.46 |
| Coronary artery disease | 0.64 | < 0.0001 |
| Osteoporotic fracture | 0.64 | 0.25 |
| Dementia | 0.64 | 0.06 |
| Intracerebral bleeding excluding contusions | 0.61 | 0.52 |
| Cardiac arrythmia | 0.6 | 0.0022 |
| Fracture of the hand | 0.56 | 0.31 |
| Number of visits to the operating theatre | 0.5 | < 0.0001 |
| Hemiplegia | 0.47 | 0.0036 |
| Fracture of the distal radius | 0.47 | 0.02 |
| Referral from our care hospital to another inpatient care proider | 0.42 | < 0.0001 |
| Cerebral infarction | 0.42 | < 0.0001 |
| Hemothorax | 0.39 | 0.13 |
| Intoxication with psychotropic substances | 0.38 | 0.01 |
| Commotio cerebri | 0.24 | < 0.0001 |
| Fracture of the scapula | 0.00002 | 0.9 |

**Appendix 2 Table J** Prognostic values of all three feature selection methods for the predictors of profit cases. Outliers were selected with the % deviation method. Results are given as area under the curve (AUC) and the corresponding 95% confidence interval (CI).

| **Methods** | **Profit Outlier Model** | | **Deficit Outlier Model** | |
| --- | --- | --- | --- | --- |
|  | **AUC** | **95% CI** | **AUC** | **95% CI** |
| Multivariable Model | 0.71 | [0.70, 0.73] | 0.67 | [0.66, 0.68] |
| Lasso | 0.69 | [0.68, 0.71] | 0.60 | [0.58, 0.61] |
| Random Forest | 0.73 | [0.72, 0.75] | 0.62 | [0.61, 0.64] |
