# Supplementary figures and images for: Predictors of High Profit and High Deficit Outliers under SwissDRG of a Tertiary Care Center
Source: PLoS One. 2015 Oct 30;10(10):e0140874. doi: 10.1371/journal.pone.0140874 (PMC4627843; doi:10.1371/journal.pone.0140874)

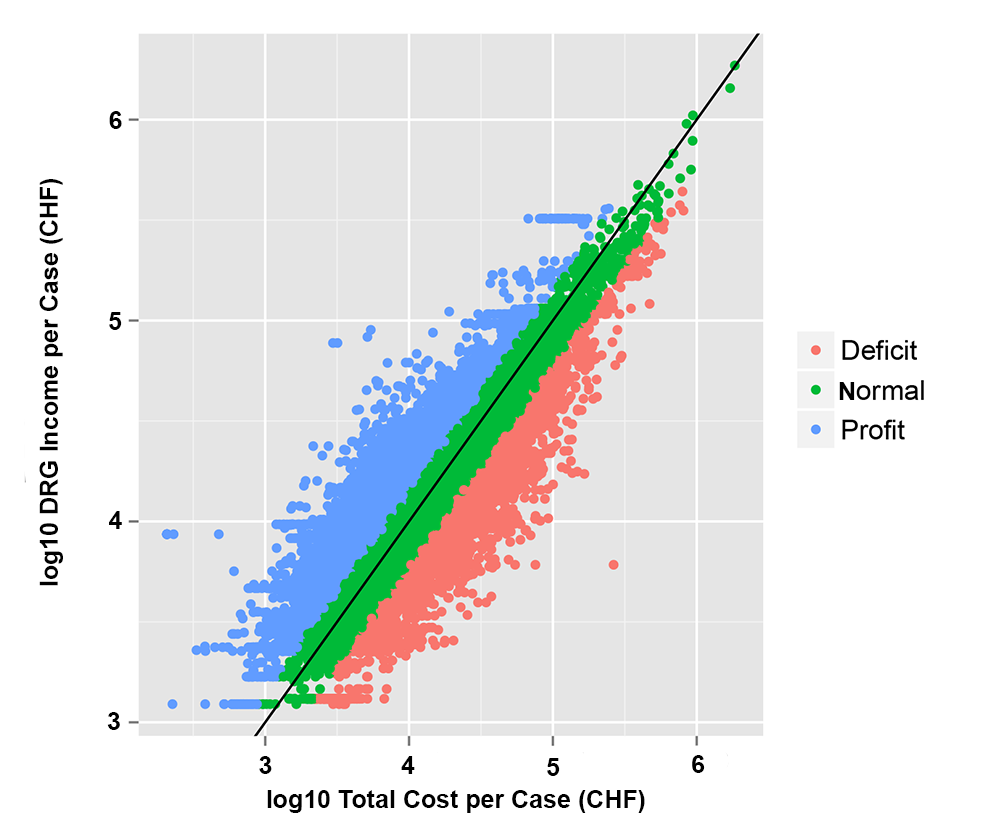

Supplement: S1 Fig — (TIF) [file pone.0140874.s003.tif]

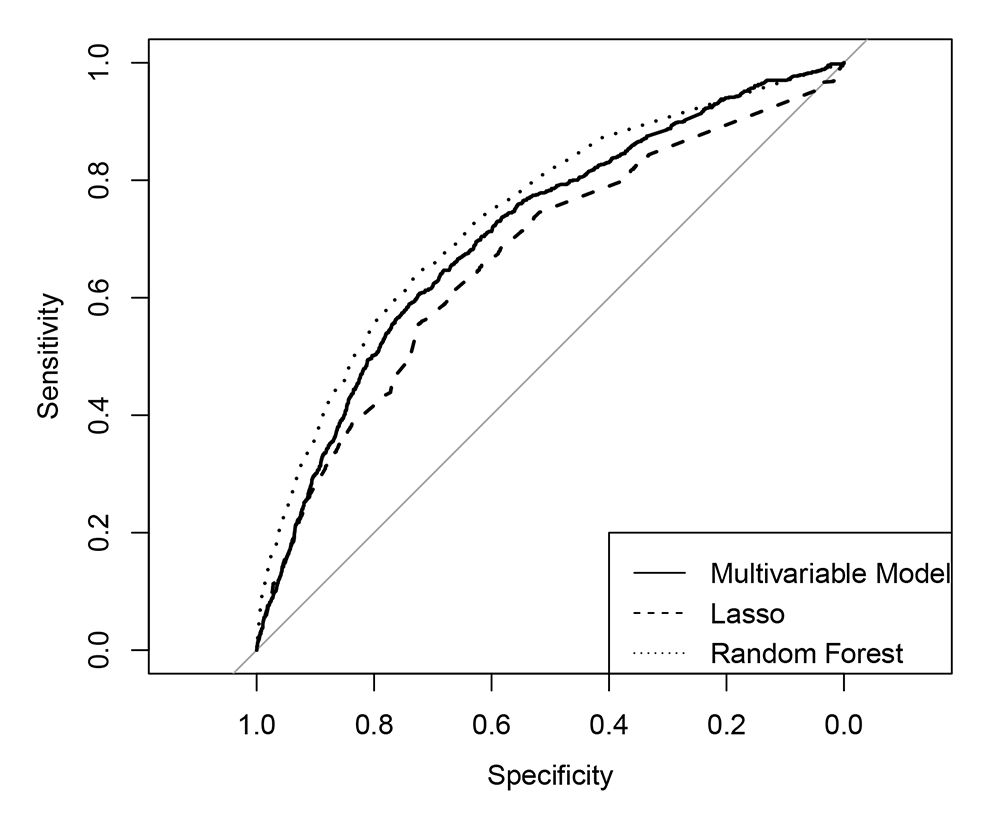

Supplement: S2 Fig — (TIF) [file pone.0140874.s004.tif]

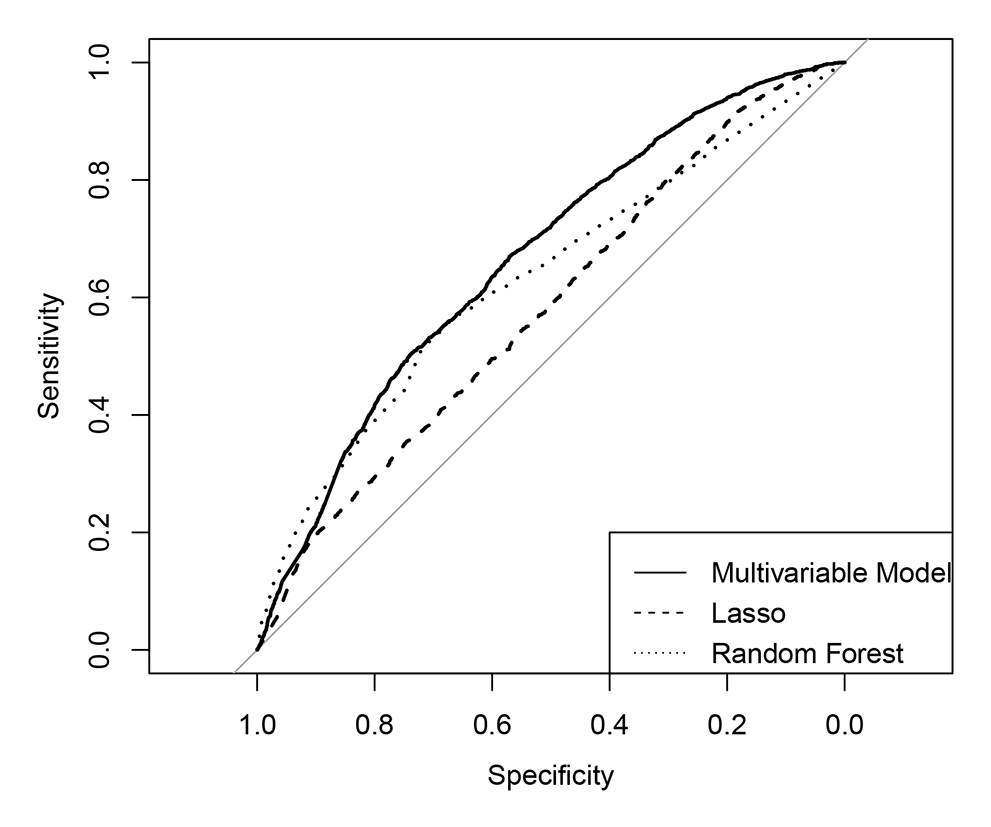

Supplement: S3 Fig — (TIF) [file pone.0140874.s005.tif]
